# Co-Occurrence of Mycotoxins in Feed for Cattle, Pigs, Poultry, and Sheep in Navarra, a Region of Northern Spain

**DOI:** 10.3390/toxins15030172

**Published:** 2023-02-22

**Authors:** Borja Muñoz-Solano, Elena González-Peñas

**Affiliations:** Department of Pharmaceutical Technology and Chemistry, Faculty of Pharmacy and Nutrition, Universidad de Navarra, 31008 Pamplona, Spain

**Keywords:** feed, LC-FLD, aflatoxins, ochratoxin A, zearalenone, deoxynivalenol, sterigmatocystin, co-occurrence

## Abstract

Mycotoxins, toxic compounds produced by fungi on raw materials, such as cereals, represent a serious health hazard. Animals are exposed to them mainly through the ingestion of contaminated feed. This study presents data about the presence and co-occurrence of nine mycotoxins: aflatoxins B1, B2, G1, and G2, ochratoxins A and B, zearalenone (ZEA), deoxynivalenol (DON), and sterigmatocystin (STER), in 400 samples of compound feed for cattle, pigs, poultry, and sheep (100 samples each) collected in Spain (2019–2020). Aflatoxins, ochratoxins, and ZEA were quantified using a previously validated HPLC method using fluorescence detection; whereas DON and STER were quantified using ELISA. Moreover, the obtained results were compared with those obtained in this country and published in the last 5 years. The mycotoxin presence in Spanish feed, especially for ZEA and DON, has been demonstrated. The maximum individual levels found were: AFB1: 6.9 µg/kg in a sample of feed for poultry; OTA: 65.5 µg/kg in a sample of feed for pigs, DON: 887 µg/kg in a sample of feed for sheep, and ZEA: 816 µg/kg in a sample of feed for pigs. Nevertheless, regulated mycotoxins appear, in general, at levels below those regulated by the EU; in fact, the percentage of samples containing concentrations above these limits was very low (from 0% for DON to 2.5% for ZEA). The co-occurrence of mycotoxins has also been demonstrated: 63.5% of the analyzed samples presented detectable levels of two to five mycotoxins. Due to the fact that the distribution of mycotoxins in raw materials can change greatly from year to year with climate conditions or market globalization, regular mycotoxin monitorization in feed is needed to prevent the integration of contaminated materials in the food chain.

## 1. Introduction

Mycotoxins are produced by filamentous fungi as secondary metabolites, mainly by the *Aspergillus*, *Penicillium*, *Fusarium*, *Claviceps,* and *Alternaria* genera. Thus far, a great number of mycotoxins have been discovered, with very different physicochemical characteristics. Among them, aflatoxins (AFs), ochratoxin A (OTA), deoxynivalenol (DON), zearalenone (ZEA), fumonisins (FUMs), and T-2/HT-2 toxins, are considered to be the most significant regarding their prevalence and/or effects on animal and human health [1,2,3,4].

The routes of exposure of animals to mycotoxins are varied, but the main one is the ingestion of contaminated feed. The toxic effects related to their ingestion, especially their chronic exposure, include carcinogenic effects, organ damage, decreased appetite, sudden death, immunosuppressive effects, acute toxicity, hormonal disorders and breeders, weak and small animal specimens, pulmonary edema in pigs, inhibition of protein synthesis, and alterations of DNA and RNA synthesis, among others [3,5,6], and the effects vary between species (i.e., ruminants are less sensitive than poultry or pigs) [5]. Therefore, mycotoxins are a major problem for animal health, especially for farm animals [5], even greater than that posed by pesticides, preservatives, or food additives [7,8].

The presence of mycotoxins in feed is not only a problem regarding animal health, but it is also a concern for human health. Mycotoxins can be present in animal-derived products such as milk, meat, and eggs, because of the possible carry-over of these toxic compounds from feed to these food products, leading to mycotoxin ingestion by humans [9,10].

Moreover, the impact of mycotoxins on the global economy is of great concern. This is due to the losses of contaminated batches and the cost of treating animal or human mycotoxicosis, the deaths of animals, or decreased animal productivity [11]. For example, the annual loss in the US corn industry, related only to high levels of AFs and the subsequent elimination of contaminated batches, is between USD 1.6 to 52.1 billion [12].

Compound feed is defined by the European Union (EU) as “a mixture of at least two feed materials, whether or not containing feed additives, for oral animal-feeding in the form of complete (sufficient for a daily ration) or complementary feed (sufficient for a daily ration only if used in combination with other feed)” [13].

Usually, the main ingredients in compound feed (hereinafter: feed) preparation are cereals [11]. The prevalence of mycotoxins in crops could be up to 60–80%, and it is supposed to be higher in the grains used for feed preparation than in food crops [14]. The infestation of crops by mycotoxigenic fungi can occur in the field, during cultivation, or during storage and processing. Inadequate agricultural and crop management practices, inadequate drying of raw materials, poor packaging, storage, and/or transport conditions, favor the growth of fungi, thus increasing the likelihood of mycotoxin contamination [15].

There are other added risk factors. First, climate change could modify fungi growth patterns and affect both the distribution and levels of mycotoxins in different parts of the world, including the appearance of mycotoxins in places where, due to climatology, their presence is expected to be limited [16,17]. For instance, increased contamination from DON and AFB1 is likely in cereals from southern Europe [9,18,19] due to a temperature increase and less water availability, especially in summer [20]. On the other hand, globalization, with its continuous growth of the markets and the improvement of the means of transporting goods, favors the worldwide distribution of raw materials used in the manufacture of feed and makes it difficult to predict feed contamination in a world zone, because imported cereals should be taken into account [3,21]. In addition, the high heterogenicity in the worldwide distribution of mycotoxins year after year [16], their chemical stability, and the difficulty of eliminating them during feed production [22], are factors that increase the serious risk of feed contamination. In fact, the analysis of several samples from different countries and zones around the world demonstrated the ubiquity of the presence of these toxic compounds in feed [16,23,24].

Moreover, the co-contamination with various mycotoxins is a very likely scenario, because cereals may be infested by different fungi, some of the fungal species can produce various mycotoxins, and different raw materials are usually mixed for feed production [4,16,21]. Even at low levels, co-occurrence can be a health problem, due to possible additivity, antagonism, or synergy of their effects [11].

For all of the above reasons, mycotoxin presence in feed is a serious problem for both feed factories and the agriculture industry, and is without easy solutions [25]. The EU, in order to guard human and animal safety, has established recommended maximum limits for some mycotoxins in feed: 900–5000 µg/kg for DON, 100–500 µg/kg for ZEA, 50–100 µg/kg for OTA, and 5000–50,000 µg/kg for FUMs (FB1+FB2) [26]. In the case of Afs, which are of great concern in the EU food and feed safety policy [17], levels have been regulated for aflatoxin B1 (AFB1) at 5–20 µg/kg [27].

However, there are other mycotoxins that are unregulated and less studied but that can also pose risks to human and animal health. For instance, sterigmatocystin (STER) is a mycotoxin structurally related to AFB1 [28]. It has been classified as group 2B (possibly carcinogenic to humans) by the International Agency of Research in Cancer (IARC) [29] and has been associated with several toxic effects, such as immunotoxicity, immunomodulatory activity, and mutagenicity [30]. For this mycotoxin, more data on its occurrence in food and feed are needed, especially due to the fact that studies of human biomonitoring of mycotoxins detected its presence in human plasma [31].

In order to control mycotoxin levels in feed, and given the difficulty of controlling their appearance, it is essential to implement correct monitoring measures for raw materials and feed [4]. These data will aid in animal and human health safeguards because they will give information regarding mycotoxin presence and co-occurrence in this matrix, they will aid to guarantee the withdrawal of contaminated feed from the food chain, and identify the use of raw materials and finished products with the lowest levels of contamination. However, in Spain very few studies regarding the presence of mycotoxins in feed have been published in the last 5 years [32,33,34] and, therefore, more information is needed to know the exposure of animals, especially to multi-mycotoxins, and to evaluate the risk that they pose to animals and humans [9].

The main objective of this study is to obtain data regarding the co-occurrence of nine mycotoxins: AFB1, aflatoxin B2 (AFB2), aflatoxin G1 (AFG1), aflatoxin G2 (AFG2), OTA, ochratoxin B (OTB), ZEA, DON, and STER, in feed prepared for four different animal species (cattle, pigs, poultry, and sheep) and collected in Spain. Moreover, a comparison is made with the data obtained in other studies carried out in this country. Due to the variability of mycotoxin contamination from year to year, only studies published in the last 5 years have been considered.

## 2. Results

**Figure 1:**
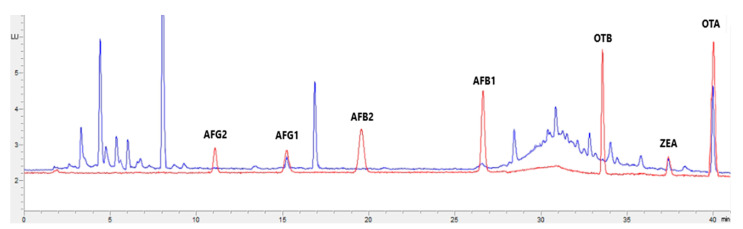
Superposition of a chromatogram of a natural feed sample for cattle (blue) with one from a calibrator at 2.5× limit of quantification (red).

**Figure 2:**
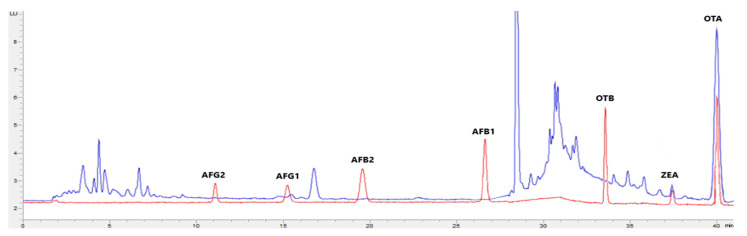
Superposition of a chromatogram of a natural feed sample for pigs (blue) with one from a calibrator at 2.5× limit of quantification (red).

**Figure 3:**
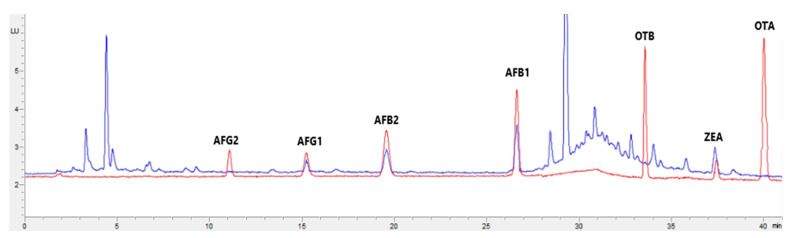
Superposition of a chromatogram of a natural feed sample for poultry (blue) with one from a calibrator at 2.5× limit of quantification (red).

**Figure 4:**
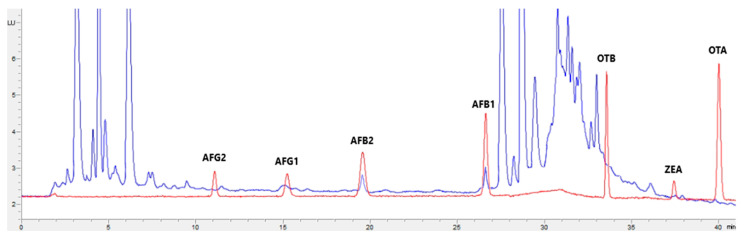
Superposition of a chromatogram of a natural feed sample for sheep (blue) with one from a calibrator at 2.5× limit of quantification (red).

Figure 1, Figure 2, Figure 3 and Figure 4 show examples of the obtained chromatograms in both calibrators and feed for different species.

### 2.1. Presence of Mycotoxins

The raw data obtained after the analysis of the 400 collected feed samples are presented in Table S6 (feed for cattle), Table S7 (feed for pigs), Table S8 (feed for poultry), and Table S9 (feed for sheep). A summary of the results, calculated as indicated in the material and methods section, is shown in Table 1, Table 2, Table 3 and Table 4.

**Figure 5:**
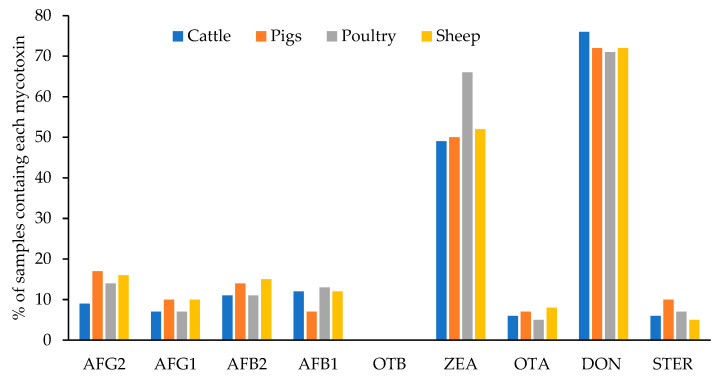
Percentage of samples contaminated with each mycotoxin in the different types of feed.

Figure 5 shows the percentage of samples of each type of feed contaminated with each mycotoxin. The 87% of the total analyzed samples (400) were contaminated with at least one mycotoxin. The percentage was very similar in the feed for the different animal species and varied between 86% and 88%: 87% for cattle and pigs, 88% for poultry, and 86% for sheep. No statistical differences (*p* < 0.05) have been encountered for the levels of each mycotoxin among different feed types. This was also previously observed by Arroyo-Manzanares et al. [34] in Spain. These authors explained that this observation was probably due to the fact that the main ingredients for all the feed tested were cereals. Only DON levels in feedstuff for pigs had a significative minor mean level than in the other feed types. Pigs are the most susceptible animals to DON [35] and the EU defined the lowest maximum level for this mycotoxin in complete feedstuff for pigs (900 µg/kg) [26]. The need to accomplish this regulation would lead to greater control by feed producers.

The most prevalent mycotoxins were ZEA and DON in all cases. These mycotoxins were detected in 49–66% and 71–76% of the samples, respectively, and depended on the type of feed. Moreover, in a low percentage of samples, other mycotoxins were detected: AFG2 (between 9–17% of samples), AFG1 (7–10%), AFB1 (7–13%), OTA (5–8%), and STER (5–7%). OTB was not detected in any feed sample.

Figure 5 shows that the highest percentage of samples contaminated with AFG2, AFG1, and STER were found in feed for pigs (17%, 10%, and 10%, respectively); AFB2 and OTA were the most prevalent in feed for sheep (15% and 8%, respectively); AFB1 and ZEA in feed for poultry (13% and 66%, respectively), and DON appeared in a higher percentage in samples of feed for cattle (76%).

Regarding the concentration levels in all feed types, the mean and median values of the levels encountered for all mycotoxins are <LOD (<LOQ for STER) of the corresponding methodologies, except for ZEA and DON. For these mycotoxins, the mean values ranged between 65.4–104.7 µg/kg and between 113.4–176.0 µg/kg, respectively. The EU has set maximum acceptable levels of ZEA in feed at 250 µg/kg for pigs and 500 µg/kg for cattle and sheep. The mean levels obtained in this study are below these maximums. However, in the case of pigs, nine samples (9%) presented levels above 250 µg/kg, with the maximum level found being 816 µg/kg. Likewise, in the case of feed for sheep, two samples (2%) presented levels above 500 µg/kg, the maximum found being 658.0 µg/kg. For DON, the EU has fixed maximum acceptable levels in feed at 5000 µg/kg and at 900 µg/kg in the case of pigs. None of the analyzed samples presented levels above these limits.

For AFB1 (EU maximum limit 20 µg/kg for pig and poultry and 5 µg/kg for dairy animals), five feed samples for sheep and one for cattle had levels higher than 5 µg/kg, the maximum levels being 6.1 µg/kg and 5.4 µg/kg, respectively.

The EU maximum limit for OTA is 50 µg/kg in feed for pigs and 100 µg/kg for poultry. Only one sample of feed for pigs exceeds these maximums (65.5 µg/kg).

### 2.2. Co-Occurrence of Mycotoxins

**Figure 6:**
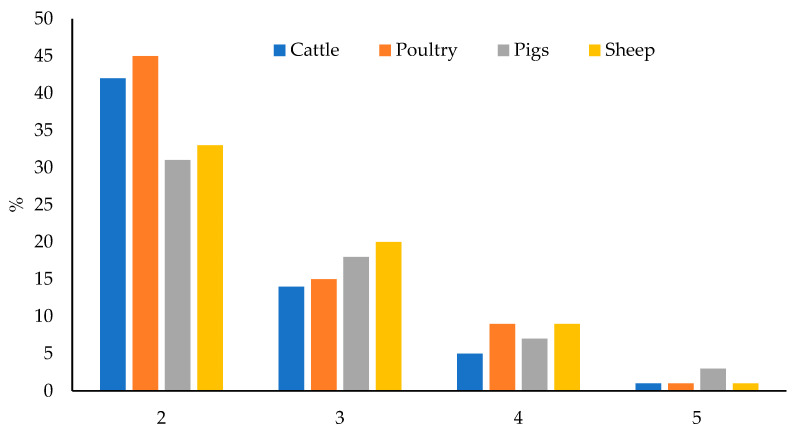
Number of co-occurring mycotoxins in each feed type.

Of the total samples, 63.5% were contaminated with two or more mycotoxins and this percentage varied between 59 and 70%, depending on the feed type. Poultry feed had the highest percentage of co-occurrence (70%); whereas feed for pigs had the lowest one (59%). Feed for sheep (63%) and cattle (62%) had similar and intermediate percentages. The maximum number of co-occurring mycotoxins was five in all feed types, in a sample of feed for poultry, sheep, and cattle; and in three samples of feed for pigs. Figure 6 shows the number of co-occurring mycotoxins in each feed type.

**Figure 7:**
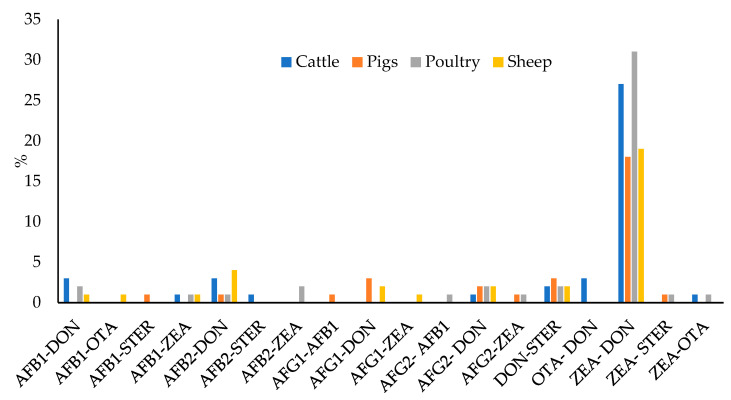
Percentage of samples containing detectable levels of two mycotoxins.

In most of the cases in which mycotoxins co-occurred, the levels of two mycotoxins were found (37.8% of total samples, 42% in feed for cattle, 31% in feed for pigs, 45% in feed for poultry, and 33% in feed for sheep). Different combinations were found, although the most recurrent one was the co-occurrence of ZEA and DON (23.8% of the 400 samples: 27% in feed for cattle, 18% in feed for pigs, 31% in feed for poultry, and 19% in feed for sheep), as can be seen in Figure 7. Correlation between the levels of two toxins was verified by means of Spearman’s rank correlation test. The correlation matrix is shown in Table S10. Significative correlation (*p* = 0.05) has been encountered between: AFG2 and AFG1 (0.2528), AFG2 and ZEA (0.1149), AFB2 and AFB1 (0.1736), AFB2 and ZEA (0.1040), and ZEA and DON (0.3129). The higher correlation factor is for ZEA and DON. This observation agrees with the fact that both mycotoxins are the most prevalent in temperate climates [14]. It is also explained that because these mycotoxins are produced by the same *Fusarium* species (F. graminearum and F. culmorum) [16,25]; whereas AFs, STER and OTA are produced by other fungi, such as *Aspergillus* in the case of AFs and STER, or *Aspergillus* and *Penicillium* in the case of OTA [25,36]. The coexistence of different fungi species in the same raw material would explain the correlation between AFs and ZEA.

Apart from ZEA–DON, the next most prevalent combinations were DON–STER, AFB2–DON, AFG2–DON, AFB1–DON, and AFG1–DON, although in all cases, the percentage of the total samples was less than 2.5%. In feed for cattle, the most prevalent were the mixtures of AFB1–DON, AFB2–DON, and OTA–DON, in all cases they were found in 3% of the samples; and in feed for pigs, AFG1–DON and DON–STER were both found in 3% of the samples. In feed for poultry, the most prevalent mixtures were AFB1–DON, AFB2–ZEA, AFG2–DON, and DON-STER, all of which appeared in 2% of the samples; and finally, in feed for sheep, the most prevalent mixture was AFB2–DON, found in 4% of the samples.

Moreover, various combinations of three mycotoxins were found in 16.8% of the 400 samples. The most frequently found was AFG2–ZEA–DON (13% of the samples) and AFB1–ZEA–DON (11% of the samples). In feed for poultry, the most prevalent mixture was AFB1–ZEA–DON (5% of the samples); whereas AFB1–ZEA–DON and AFG2–ZEA–DON were the most prevalent mixtures in feed for sheep (all in 4% of the samples). In the case of feed for pigs, 4% of the samples contained levels of AFG2–ZEA–DON; and, for cattle, 3% of the samples presented AFB1–ZEA–OTA.

In addition, mixtures of four mycotoxins were found in 7.3% of the total samples, and the most prevalent one was AFG2–AFG1–ZEA–DON (2% of all samples and 2% of each type of feed). Moreover, the mixture AFG1–AFB2–ZEA–DON is the most prevalent in feed for pigs (5% of the samples of this feed type).

Finally, 1.5% of the total samples contained detectable levels of five mycotoxins. Especially in feed for pigs (3% of the samples). The most prevalent mixture was AFB2–AFB1–ZEA–DON–STER in 0.5% of the total samples.

Regarding other AFs, in 38 samples (9.5% of all the samples) there was the co-occurrence of two or three of them. The most frequent mixture found was AFB2–AFB1 in four samples, and AFG2–AFG1 and AFG2–AFB2 in three samples each. The sum of the levels of AFs in feed for poultry and pigs was always less than 20 µg/kg, the maximum level legislated for AFB1 in feed [27]: 13.0 µg/kg in a sample of feed for poultry and 8.6 µg/kg in a sample of feed for pigs. In the case of dairy animals, seven samples surpassed the value of 5 µg/kg, the maximum level legislated for AFB1 in feed for dairy animals [27]: three samples of feed prepared for sheep (7.1–14.2 µg/kg) and four samples of feed for cattle (5.0–8.1 µg/kg).

## 3. Discussion

The study of the presence of mycotoxins in feed is a subject of great interest in order to safeguard animal and human health as well as the global economy. This is due to the possible appearance of dangerous contaminants, among them mycotoxins, as has been demonstrated by different authors. In fact, Gruber-Dorninger et al. 2019 [16], after analyzing finished feed and different raw materials, demonstrated that the highest prevalence of mycotoxins was in feed. For this reason, studies for the detection and quantification of these toxic compounds in this matrix are needed [16]. During the last 5 years, some studies on the presence of AFB1 and total AFs, as well studies on the presence of OTA, DON, ZEA, STER [16,34,37,38,39,40,41], and others, such as enniatins or beauvericin, [34,42] in feed can be found. These studies are generally limited to a specific world region and/or a low number of samples, although some of them have been carried out worldwide [16]. Globally, AFs, DON, and ZEN were the mycotoxins most studied from 2016 to 2018 [4].

One of the most extensive studies is the one elaborated by Gruber-Dorninger et al. [16]. These authors collected a total of 74,821 samples (21,588 of them from feed and 1463 from southern Europe countries) over a period of 10 years from 100 different countries. Of them all, 88% samples (feed and many other raw materials used in animal nutrition) contained at least one toxin. In the case of feed, the most prevalent were FUMs (73%), DON (70%), and ZEA (56%). AFB1, OTA, and T-2 were also detected, although in lower percentages (<30%). These authors demonstrated differences in the presence of mycotoxins among different world zones. In southern Europe, the most prevalent mycotoxins were FUMs (74.9%), DON (52.9%), and ZEA (36.3%). AFB1 was also found in samples at a higher incidence (28.9%) than in the rest of Europe, having 7.4% and 2.1% of the samples exceeding 5 or 20 µg/kg, respectively (EU maximum limits). Moreover, in samples of feed, the co-occurrence of mycotoxins was demonstrated. The most frequently encountered combinations were: DON-ZEA, DON-FUMs (both 48%), and ZEA-FUM (43%) of the feed samples analyzed worldwide.

In Spain, very few studies have been published in the last 5 years (2018–2022). Romera et al. [32] published a study in 2018 regarding the presence of 15 mycotoxins, among them, AFs, OTA, ZEA, and DON, in 52 samples of feed for cattle (6), pigs (20), poultry (9), and sheep (17) (among others), collected between 2012 and 2014. Moreover, Arroyo-Manzanares et al. [34] studied the presence of 19 mycotoxins in 226 samples of feed for pigs collected in 2017 from different farms and suppliers in Spain. Bervis et al. [33] studied the presence of AFB1 in 22 feed samples intended for cattle and collected in Spain between 2015–2016.

In the present study, 400 samples of feed for cattle, pigs, poultry, and sheep (100 for each) collected in Navarra were analyzed for AFs, OTA, OTB, ZEA, STER, and DON.

Navarra, a region in the north of Spain between the Pyrenees and the Ebro River, has a varied climate. Cereal-producing areas (barley, wheat, and oat) are characterized by dry summers and winters, spring and autumn rains, and cold winters. The average annual precipitation is between <400 to 1000 L/m^2^ and the mean annual temperature is between 12 and >14 °C. Corn is only grown in the north, with a temperate climate (average annual temperature of 12–14 °C), but in this area the mean annual rainfall is higher: 1000–2500 L/m^2^ [43].

### 3.1. Aflatoxins

In the present study, levels of AFB1 have been found in 7% of samples in feed for pigs in a region of northern Spain, with levels up to 6.2 µg/kg. Additionally, in this country, Arroyo-Manzanares et al. (2019) [34] encountered 3% of positive samples for AFB1 in feed for pigs, containing between 0.29–2.91 µg/kg of this mycotoxin. Romera et al. [32] detected AFB1 in three samples (15%) in this type of feed, but with levels <LOQ. The maximum AFB1 regulated level in feeds for pigs in the EU is 20 μg/kg.

In the other types of feed (cattle, poultry, and sheep) we have found a higher AFB1 prevalence: 12–13% with samples exceeding the 5 μg/kg level: five feed samples for sheep and one for cattle. Bervis et al. [33] studied the presence of AFB1 in feed intended for cattle. This mycotoxin was detected in 86% of feed and two samples lightly exceeded the maximum AFB1 regulated levels in feeds for dairy cows, established at 5 μg/kg [27]. Romera et al. [32] found AFB1 in four samples but with levels <LOQ.

Therefore, only 8 of the 700 analyzed feed samples in Spain (1.1%) had an AFB1 value higher than that regulated by the EU, and the AFB1 occurrence in Spain during 2012–2020 (years in which samples were collected) is lower than that found in the southern European region by Gruber-Dorninger et al. (28.9%) [16].

Other AFs found in feed samples in Spain by Arroyo-Manzanares et al. [34] were AFB2 (1.32%) and AFG1 (0.88%) with levels between 0.22 and 1.06 µg/kg; AFG2 was not detected in any sample. Romera et al. [32] did not find AFs levels higher than 4 µg/kg (LOQs for AFB2, AFG1, AFG2) in any of the feed types studied. In the present study, both the prevalence for AFB2 (12.8%), AFG1 (8%), and AFG2 (14%) and the levels found are higher (between <LOD–6.5 µg/kg).

### 3.2. Ochratoxin A

In the present study, levels of OTA were found in all types of feed with a prevalence between 5–8% and a maximum value of 65.5 µg/kg in a sample of feed for pigs, which was the only one that surpassed the guidance value in the EU (50 µg/kg). Feed for cattle had the lowest value regarding both the mean of positive samples and maximum level; whereas feed for sheep and pigs presented the highest levels. Arroyo-Manzanares et al. 2019 [34] did not detect OTA > 2.5 µg/kg in any sample. Romera et al. [32] detected OTA in 16 samples (30.7%), but the OTA levels were not quantifiable in any of the analyzed samples of feed for cattle, pigs, poultry, or sheep (LOQ 25 µg/kg). These differences are not unexpected, because the levels of OTA in cereals vary greatly among regions and cereal types [44]. Therefore, only one of the 678 feed samples analyzed in Spain (0.15%) had OTA levels that surpassed the maximum level established by the EU of 50 µg/kg [26].

### 3.3. Zearalenone

Quantifiable levels of ZEA were found in all types of feed analyzed in the present study in 49–66% of the samples. Maximum levels were from 413 to 816 μg/kg and 11 samples surpassed the EU guidance values. Arroyo-Manzanares et al. [34] found this mycotoxin in 7.02% of the samples of feed for pigs at levels between 101 and 956 µg/kg, and six samples surpassed the guidance levels for pig feed. Romera et al. [32] detected ZEA in seven samples (13.5%). In three of them, levels were higher than the LOQ of the method between 54.5 and 104.4 μg/kg. This highest level was found in a sample of feed for sheep. In the EU, the guidance levels for ZEA in feed in animals are between 100–500 µg/kg, regarding different types of feed. Therefore, from the 678 analyzed samples in Spain, only 17 samples surpassed the guidance levels (2.5%).

### 3.4. Deoxynivalenol

For DON, none of the analyzed samples in the present study had levels higher than those indicated by the EU [26]. Arroyo-Manzanares et al. [34] found this mycotoxin in 4.39% of the samples in values from 153–555 μg/kg, and Romera et al. [32] detected DON in five samples, but only two had quantifiable levels: 254.9 and 289.9 µg/kg. Therefore, none of the 678 samples analyzed in Spain had levels higher than those regulated by the EU.

### 3.5. Sterigmatocystin

Regarding STER levels, a mycotoxin considered a possible human carcinogen (group 2B) according to the IARC classification [29], very few studies of occurrence in animal feed have been carried out, so it is very difficult to establish a relationship between the levels found in the present study and others studies in Spain. Only Arroyo-Manzanares et al. [34] studied the presence of this mycotoxin in feed for pigs with a prevalence of 2.19% and values between 11.2 and 308 µg/kg. In the present study, STER was detected in all types of feed, with a prevalence between 5–10% of the samples with maximum values from 4.7 and 6.1 µg/kg. The knowledge about the presence of this mycotoxin in feed, and also in food, should be improved because it has been detected in plasma samples in human biomonitoring studies [31].

Table 5, Table 6, Table 7, Table 8 and Table 9 show a summary of the levels of mycotoxins encountered in feed samples from Spain. Due to the low percentage of samples with mycotoxin levels above the established limits in the EU, Spanish feed, at least during the years of collection of the analyzed samples, did not suppose a high risk for animal and human health. However, the monitorization of the most important mycotoxins, as well as others that are less known (such emerging mycotoxins), should be maintained because the distribution and presence of mycotoxins could change from year to year, as has been previously explained.

### 3.6. Co-Occurrence

As has been explained above, feed and raw materials destined for animal consumption are frequently contaminated with multiple mycotoxins. In fact, the simultaneous presence of mycotoxins is the most likely scenario in feed [16].

In the worldwide study conducted by Gruber-Dorninger et al. (2019) [16], 64% of the samples analyzed contained at least two different mycotoxins. For feed, the combinations of DON, FUMs, and ZEA were the most frequently found: DON-ZEA and DON-FUMs, both in 48% of the samples, and ZEA-FUMs in 43%.

The data obtained in the present study also demonstrate the co-contamination in feed samples in Spain. Of the total samples, 63.5% presented detectable levels between 2–5 mycotoxins in all types of feed. The most frequent combination was DON-ZEA in 23.8% of all samples.

In Spain, other authors have also demonstrated co-occurrence. Arroyo-Manzanares et al. [34] found more than one mycotoxin in 98.7% of the samples of feed for pigs; moreover, 2.19% contained up to eight toxins. Romera et al. [32] found one sample of cattle feed (16.7% of cattle feed), which contained simultaneously ZEA and DON at quantifiable levels (88.2 and 289.9 μg/kg, respectively).

Therefore, and as expected, the most common combination was DON-ZEA, but in a lower percentage than that found by Gruber-Dorninger et al. (2019) [16].

## 4. Conclusions

Chronic ingestion of mycotoxins, such as ZEA, DON, or AFB1 among others, has been related to serious health effects on animals. Also, these toxic compounds can appear in derived animal food, such as milk, eggs, or meat, being also a problem for human health. Moreover, feed contamination is a worldwide economic problem, due to crop refusal or the cost of treating animal or human mycotoxicosis.

The present study demonstrates the contamination of feed for cattle, poultry, pigs and sheep in a region of northern Spain (Navarra) with these toxic substances. No statistical differences have been encountered for mycotoxin levels regarding the type of feed. The maximum levels found were: AFB1: 6.9 µg/kg in a sample of feed for poultry; OTA: 65.5 µg/kg in a sample of feed for pigs, DON: 887 µg/kg in a sample of feed for sheep, and ZEA: 956 µg/kg in a sample of feed for pigs. Regulated mycotoxins appear, in most of the cases, at levels below the established by the EU. The most prevalent were DON (73% of the total samples) and ZEA (73% and 54% of the total samples, respectively) as expected by the results in other studies in Spain and around the world, due to the temperate climate of Navarra and because both mycotoxins are produced by the same *Fusarium* species.

During the years of collection of the analyzed samples from Spain (2012–2020), the percentage of samples that have surpassed the levels indicated by the EU was very low; in fact, lower than that would be supposed for a country from southern Europe. However, it is known that the distribution and presence of mycotoxins could change year to year due to variations in climatic conditions or the global trade of raw materials. For this reason, programs of monitorization of the most important mycotoxins, as well as others that are less known (such as emerging mycotoxins) in feed should be repeatedly carried out in order to keep mycotoxin levels under control and to prevent the incorporation of contaminated raw materials in the food and feed supply chain.

Moreover, the co-occurrence of mycotoxins in feed has been demonstrated and 63.5% of the samples presented detectable levels of 2–5 mycotoxins. Although the levels were low, the knowledge regarding their combined effects on animal and human health should be increased.

## 5. Materials and Methods

### 5.1. Chemical and Reagents

The reagents used in the chromatographic separation of mycotoxins and their extraction from feed samples were: acetonitrile (ACN) and methanol (MeOH), both of gradient grade and purchased from Honeywell Riedel-de Haën (Muskegon, MI, USA); chloroform and water, also liquid chromatography (LC) gradient grade, provided by Fisher Scientific (Bishop Meadow, UK); and pro-analysis grade orthophosphoric acid obtained from Panreac (Barcelona, Spain). In addition, OASIS PRIME HLB SPE cartridges were obtained from Waters (Milford, MA, USA). In the case of DON and STER analysis, commercial enzyme-linked immunosorbent assay (ELISA) kits suitable for mycotoxin quantification in feed were used. For DON analysis, the RIDASCREEN^®^ FAST DON SC kit (Art. No. R5905 R-Biopharm AG, Darmstadt, Germany) was employed; while for STER quantification, the Creative-diagnostic Sterigmatocystin DEIANJ08 ELISA kit was selected (NY, USA). In both cases, all the reagents needed were included in the kits.

### 5.2. Mycotoxin Standards

AFB1 2 μg/mL (PubChem CID: 186907), AFB2 0.5 μg/mL (PubChem CID: 2724360), AFG1 2 μg/mL (PubChem CID: 14421), AFG2 0.5 μg/mL (PubChem CID: 2724362), OTA (PubChem CID: 442530), OTB 10 µg/mL (PubChem CID: 20966), and ZEA 100 µg/mL (PubChem CID: 5281576) were obtained from Sigma-Aldrich (Merck KGaA, Darmstadt, Germany). All mycotoxins (purity greater than 98%) were obtained in a ready-to-use dissolution in ACN, with the exception of OTA. This mycotoxin was purchased in powder form and dissolved in MeOH in order to obtain a concentration of 1 mg/mL. The true concentration of the resulting OTA solution was verified through ultraviolet spectrophotometry at 333 nm (UVIKON 922, Kontron Instruments SA, Madrid, Spain). All solutions were stored at −20 °C until use.

### 5.3. Safety Precautions

Mycotoxins are toxic compounds for humans and, therefore, some safety precautions were considered to guarantee the security of the analysts. All mycotoxin standards were used as a solution, including OTA. In this case, its previous dissolution was carried out by injecting the solvent (MeOH) directly into the sealed vial through its septum, thus avoiding the weighing of OTA powder and the formation of aerosols. Other personal protection measures were in use during dissolution and sample handling, such as the use of gloves, masks, and a laminar flow hood. In order to avoid the degradation of photosensible mycotoxins, work was always carried out in low light conditions.

### 5.4. Animal Feed Samples

During 2019 and 2020, a total of 400 samples of feed were collected from different feed factories located in Navarra (northern Spain). Of them, 100 were formulated for cattle feed, 100 for pigs, 100 for poultry, and 100 for sheep. All samples came from different production batches. Approximately 250 g was collected from each batch, and all samples were stored in a freezer at −20 °C until processing and analysis. The selected feeds were prepared, in all cases, based on cereals. Their moisture content was <12%.

### 5.5. Mycotoxin Solutions and Calibrators Preparation

A stock solution containing AFG2, AFB2, AFG1, AFB1, OTB, OTA, and ZEA was initially prepared. To do this, the necessary volume of each mycotoxin standard solution was diluted with ACN to obtain concentrations of 12.6 μg/L for AFG2 and AFB2, 40 μg/L for AFG1 and AFB1, 100 μg/L for OTB and OTA, and 840 μg/L for ZEA. The final volume of this stock solution was 100 mL. Afterward, and in order to prepare the different calibrators for the calibration curves, an aliquot of the stock solution was taken and kept in the dark for 30 min until the solution reached room temperature; afterward, a given volume of the stock solution was evaporated until dry using a vacuum evaporator at 65 °C (Genevac Mivac Duo, Ipswich, UK). The resulting residue was dissolved in 5 mL of a mixture replicating the initial chromatographic mobile phase conditions, consisting of 70% water (acidified with 0.1% orthophosphoric acid), 21% MeOH, and 9% ACN.

For the quantification of DON and STER by ELISA using commercial kits, calibrators were included in the kits and no preparation was needed.

### 5.6. Sample Preparation

For complete homogenization, 100 g of feed was crushed twice in an analytical mill (A 11 basic from IKA^®^-Werke GmbH & Co. KG, Staufen, Germany) until the sample had a flour-like appearance.

For AFG2, AFG1, AFB2, AFB1, OTB, OTA, and ZEA quantification, a previously validated LC-FLD method was employed [45]. It is briefly described below.

From each feed sample, 0.5 g was weighed into a conical tube (15 mL) and 5 mL of a solution of ACN/water/orthophosphoric acid (79/20/1) was added. The mixture was then vortexed (Fisherbrand™ Digital Multi-Tube Vortexer, Fisher Scientific, Bishop Meadow, UK) for 1 h, followed by centrifugation at 5500 revolutions per minute (rpm) for 10 min. Afterwards, to perform a first cleanup of the extract, 2 mL of the supernatant was passed through an Oasis PRIME HLB SPE cartridge in pass-through mode (Waters, Milford, MA, USA). Subsequently, 0.5 mL of the eluate and 0.5 mL of chloroform were placed in another conical tube (15 mL) and vortexed for 10 s. Later, and with the help of a loaded syringe, 5 mL of LC gradient-grade water was rapidly injected to achieve a dispersion of the chloroform into the water, forming a cloudy emulsion. The mixture was vortexed once more for 30 s and centrifuged at 7000 rpm for 5 min to separate the chloroform phase, containing the mycotoxins. The supernatant was removed, then 200 μL of chloroform was taken and evaporated in a 1.5 mL Eppendorf tube at 65 °C in a Genevac Mivac Duo concentrator. Before the chromatographic analysis, the residue was redissolved in 200 μL of the initial mobile phase and, finally, filtered (0.45 μm) before being transferred to the vial for chromatographic analysis.

For the quantification of DON and STER, commercial ELISA kits were used following the instructions recommended by the manufacturer.

For the DON analysis, the procedure was based on extraction in distilled water. Five grams of the homogenized sample was weighed and mixed with 100 mL of distilled water in a suitably closed container. Then, it was vigorously shaken manually for 3 min, and the extract was filtered through a Whatman No. 1 filter, obtaining the final eluate to be used in the determination.

For the STER analysis, the sample was ground to pass a 20-mesh sieve and mixed thoroughly prior sub-sampling. Then, 50 g of grounded sample and 5 g of NaCl were transferred to a beaker. A total of 100 mL of a solution containing MeOH (80%)/water was added and mixed for 1 min at high speed. Next, a minimum of 10 mL was filtered through a Whatman No. 1 filter; then, 5 mL of the eluate was diluted with 20 mL of water and the mixture was shaken.

### 5.7. Mycotoxin Analytical Methods

Chromatographic analysis was carried out as previously described in [45]. The equipment used was an LC infinity II (Agilent Technologies, Santa Clara, CA, USA) coupled to an FLD detector. The column was a C18 Cortecs T3 (Waters, Milford, MA, USA), 150 mm × 4.6 mm × 2.7 μm, linked to a C18 Cortecs T3 precolumn (Waters, Milford, MA, USA) of 5 mm × 3.9 mm × 2.7 μm. The photoderivatization of AFB1 and AFG1 was performed between the column and the detector using a photochemical reactor for the derivatization of AFs with UV light (UVE, LcTech, Dorfen, Germany) at 254 nm. The chromatographic mobile phase consisted of acidified water (0.1% orthophosphoric acid), ACN, and MeOH in gradient conditions. Chromatographic separation was achieved at a flow rate of 1.4 mL/min. The injection volume was 40 μL and the column temperature was set at 40 °C. After separation, 6 min were required for column re-equilibration (post-time). The FLD conditions for AFs were: λ_excitation_ 365 nm and λ_emission_ 440 nm; whereas for OTA, OTB, and ZEA were: λ_excitation_ 234 nm and λ_emission_ 469 nm.

The developed methodology was validated in four matrices: feed for cattle, poultry, pigs, and sheep, following the EU guidelines [46,47,48]. Recovery was calculated at three concentration levels in intermediate conditions (3 days) and the values obtained ranged between 73.6% (AFG1 in poultry feed) and 88.0% (AFB2 in feed for pigs), with relative standard deviation (RSD) (%) values of less than 7% in each one of the feed types. The recovery values have been applied as a correction factor for mycotoxin quantification in the samples. The values of the LODs and LOQs (for which precision and linearity were assessed) are indicated in Table 10.

The obtained values for LOQs are well below the maximum levels fixed in feed by the EU (100–500 µg/kg for ZEA, 50–100 µg/kg for OTA, and 5–20 µg/kg for AFB1 [26,27], and this methodology was adequate enough for the monitorization of the selected mycotoxins in feed.

For analyzing DON and STER, the instructions provided by the suppliers of the commercial ELISA kits were followed. To do this, the reagents included in the kits were maintained at 2–8 °C and, before use, they were brought to room temperature and shaken. In addition, the necessary microwells were separated and tempered.

For the quantification of both mycotoxins, 50 μL of a standard solution, or a prepared sample, was added to each corresponding well using a pipette. Afterwards, 50 μL of the corresponding enzyme conjugate, and in the case of DON, 50 μL of anti-mycotoxin antibody solutions, was added to the bottom of each well and, after mixing, the plate was incubated: 5 min at room temperature for DON and 30 min at 37 °C for STER. After this time, the content of each well was poured into a clean filter towel and the well was washed with 250 μL of the corresponding buffer washing solution, 3–5 times for 10 s each. Later, the corresponding substrate/chromogen was added and, after mixing, it was incubated again as indicated above. Finally, 100 μL of the stop solution for DON and 50 μL in the case of STER were added to each well and the absorbance at 450 nm for both mycotoxins was measured.

The ELISA kits provided standard solutions that were treated along with the samples. Mycotoxin concentration was calculated using the standard curves obtained in each case. LOQs for DON and STER were 74 µg/kg and 0.8 µg/kg, respectively, and concentration ranges were 74–6000 µg/kg for DON and 0.8–200 µg/kg for STER.

### 5.8. Analysis of Samples

The 100 samples from each animal species were analyzed in a unique sequence in which calibrators were included in order to obtain the calibration curves for mycotoxin quantification. Each sequence was accepted if the calibration curve satisfied the criteria defined during method validation: at least six concentration levels, an R^2^ value > 0.99, and a relative error (in percentage) less than 15% between the back-calculated mycotoxin concentration obtained in calibrators and their nominal value. The obtained calibration curves are presented in Tables S1–S4. Moreover, the retention time for each mycotoxin was registered in samples and in calibrators, and every value in the samples was compared with the mean value obtained for this mycotoxin in the calibrators. The values should not differ by more than 2.5% (Table S5).

### 5.9. Treatment of Results

Levels higher than the limit of detection (LOD) and lower than the limit of quantification (LOQ) were considered for the analysis of the results. For those samples with a mycotoxin level lower than the respective LOD, the value used was 0. The STATA/IC 12.0 program was used for statistical analysis of variance (ANOVA) in order to compare levels of each mycotoxin among different types of feed. Correlation between the levels of two toxins was tested by means of Spearman’s rank correlation test. For statistical significance, a probability value of 0.05 was used.

## Figures and Tables

**Table 1:** Results obtained in samples of feed for cattle.

Parameter	AFG2	AFG1	AFB2	AFB1	OTB	ZEA	OTA	DON	STER
% Positive (>LOD)	9.0	7.0	11.0	12.0	0	49.0	6.0	76.0	6.0
Mean positive samples (µg/kg)	1.7	*2.9* *	1.5	*3.4*	<LOD *	133.4	*6.3*	177.8	3.3
Mean (µg/kg)	<LOD	<LOD	<LOD	<LOD	<LOD	*65.4*	<LOD	135.1	<LOQ *
Median (µg/kg)	<LOD	<LOD	<LOD	<LOD	<LOD	<LOD	<LOD	79.3	<LOQ
1st Quartile (µg/kg)	<LOD	<LOD	<LOD	<LOD	<LOD	<LOD	<LOD	123.0	<LOQ
3rd Quartile (µg/kg)	<LOD	<LOD	<LOD	<LOD	<LOD	98.7	<LOD	189.0	<LOQ
Maximum (µg/kg)	3.3	3.4	3.2	5.4	<LOD	413.0	*7.7*	574.0	4.7

* LOD: limit of detection. LOQ: limit of quantification Italics: Level >LOD, <LOQ.

**Table 2:** Results obtained in samples of feed for pigs.

Parameter	AFG2	AFG1	AFB2	AFB1	OTB	ZEA	OTA	DON	STER
% Positive (>LOD)	17.0	10.0	14.0	7.0	0	50.0	7.0	72.0	10.0
Mean positive samples (µg/kg)	1.9	4.1	2.1	5.3	<LOD	162.3	22.9	157.5	3.6
Mean (µg/kg)	<LOD *	<LOD	<LOD	<LOD	<LOD	81.2 *	<LOD	113.4	<LOQ *
Median (µg/kg)	<LOD	<LOD	<LOD	<LOD	<LOD	<LOD	<LOD	<LOQ	<LOQ
1st Quartile (µg/kg)	<LOD	<LOD	<LOD	<LOD	<LOD	27.0	<LOD	101.0	<LOQ
3rd Quartile (µg/kg)	<LOD	<LOD	<LOD	<LOD	<LOD	88.4	<LOD	162.8	<LOQ
Maximum (µg/kg)	4.4	6.0	3.9	6.2	<LOD	816.0	65.5	410.0	6.1

* LOD: limit of detection. LOQ: limit of quantification. Italics: Level >LOD, <LOQ.

**Table 3:** Results obtained in samples of feed for poultry.

Parameter	AFG2	AFG1	AFB2	AFB1	OTB	ZEA	OTA	DON	STER
% Positive (>LOD)	14.0	7.0	11.0	13.0	0	66.0	5.0	71.0	7.0
Mean positive samples (µg/kg)	2.0	4.3	1.7	5.0	<LOD *	150.0	18.1	255.3	3.4
Mean (µg/kg)	<LOD	<LOD	<LOD	<LOD	<LOD	99.0	<LOD	176.0	<LOQ *
Median (µg/kg)	<LOD	<LOD	<LOD	<LOD	<LOD	*70.5* *	<LOD	178.0	<LOQ
1st Quartile (µg/kg)	<LOD	<LOD	<LOD	<LOD	<LOD	<LOD	<LOD	<LOQ	<LOQ
3rd Quartile (µg/kg)	<LOD	<LOD	<LOD	<LOD	<LOD	144.5	<LOD	259.8	<LOQ
Maximum (µg/kg)	3.9	5.6	3.1	6.9	<LOD	489.0	23.2	755.0	5.1

* LOD: limit of detection. LOQ: limit of quantification. Italics: Level >LOD, <LOQ.

**Table 4:** Results obtained in samples of feed for sheep.

Parameter	AFG2	AFG1	AFB2	AFB1	OTB	ZEA	OTA	DON	STER
% Positive (>LOD)	16.0	10.0	15.0	12.0	0	52.0	8.0	72.0	5.0
Mean positive samples (µg/kg)	1.9	4.3	2.0	4.4	<LOD *	201.3	21.3	238.3	3.7
Mean (µg/kg)	<LOD	<LOD	<LOD	<LOD	<LOD	104.7	<LOD	171.5	<LOQ *
Median (µg/kg)	<LOD	<LOD	<LOD	<LOD	<LOD	55.5 *	<LOD	130.0	<LOQ
1st Quartile (µg/kg)	<LOD	<LOD	<LOD	<LOD	<LOD	<LOD	<LOD	<LOQ	<LOQ
3rd Quartile (µg/kg)	<LOD	<LOD	<LOD	<LOD	<LOD	158.3	<LOD	287.8	<LOQ
Maximum (µg/kg)	4.0	6.5	4.9	6.1	<LOD	658.0	45.3	887.0	5.6

* LOD: limit of detection. LOQ: limit of quantification. Italics: Level >LOD, <LOQ.

**Table 5:** Results obtained in Spanish samples of feed for cattle.

Mycotoxin	Total Samples	Positives	Percentage of Positives	Max. Value Found µg/kg	Samples Collection	Year of Publication	Reference
**AFB1**	100	12	12	5.4	2019–2020	-	This study
	6	2	33.3	<2	2012–2014	2018	[32]
	22	19	86	5.2	2015–2016	2021	[33]
**Total**	**128**	**33**	**25.8**	**5.4**	**2012–2020**		
**AFB2**	100	11	11	3.2	2019–2020	-	This study
	6	0	0	<4	2012–2014	2018	[32]
**Total**	**106**	**11**	**10.4**	**3.2**	**2012–2020**		
**AFG1**	100	7	7	3.4	2019–2020	-	This study
	6	0	0	<4	2012–2014	2018	[32]
**Total**	**106**	**7**	**6.6**	**3.4**	**2012–2020**		
**AFG2**	100	9	9	3.3	2019–2020	-	This study
	6	0	0	<4	2012–2014	2018	[32]
**Total**	**106**	**9**	**8.5**	**3.3**	**2012–2020**		
**DON**	100	76	76	574	2019–2020	-	This study
	6	1	16.7	289.9	2012–2014	2018	[32]
**Total**	**106**	**77**	**72.6**	**574**	**2012–2020**		
**OTA**	100	6	6	7.7	2019–2020	-	This study
	6	2	33.3	<25	2012–2014	2018	[32]
**Total**	**106**	**8**	**7.5**	**7.7**	**2012–2020**		
**STER**	100	6	6	4.7	2019–2020	-	This study
**Total**	**100**	**6**	**6**	**4.7**	**2019–2020**		
**ZEA**	100	49	49	413	2019–2020	-	This study
	6	1	16.7	88.2	2012–2014	2018	[32]
**Total**	**106**	**50**	**47.2**	**413.0**	**2012–2020**		

**Table 6:** Results obtained in Spanish samples of feed for pigs.

Mycotoxin	Total Samples	Positives	Percentage of Positives	Max. Value Found µg/kg	Samples Collection	Year of Publication	Reference
**AFB1**	100	7	7	6.2	2019–2020	-	This study
	226	7	3.1	2.9	2017	2019	[34]
	20	3	15	<2	2012–2014	2018	[32]
**Total**	**346**	**17**	**4.9**	**6.2**	**2012–2020**		
**AFB2**	100	14	14	3.9	2019–2020	-	This study
	20	0	0	<4	2012–2014	2018	[32]
	226	3	1.3	1.1	2017	2019	[34]
**Total**	**346**	**17**	**4.9**	**6.2**	**2012–2020**		
**AFG1**	100	10	10	6	2019–2020	-	This study
	226	2	0.9	0.4	2017	2019	[34]
	20	0	0	<4	2012–2014	2018	[32]
**Total**	**346**	**12**	**3.5**	**6**	**2012–2020**		
**AFG2**	100	17	17	4.4	2019–2020	-	This study
	226	0	0		2017	2019	[34]
	20	0	0	<4	2012–2014	2018	[32]
**Total**	**346**	**17**	**4.9**	**4.4**	**2012–2020**		
**DON**	100	72	72	410	2019–2020	-	This study
	226	10	4.4	555	2017	2019	[34]
	20	1	5	254.9	2012–2014	2018	[32]
**Total**	**346**	**83**	**23.9**	**4.4**	**2012–2020**		
**OTA**	100	7	7	65.5	2019–2020	-	This study
	226	0	0		2017	2019	[34]
	20	8	40	<25	2012–2014	2018	[32]
**Total**	**346**	**15**	**4.3**	**65.5**	**2012–2020**		
**STER**	100	10	10	6.1	2019–2020	-	This study
	226	5	2.2	308	2017	2019	[34]
**Total**	**326**	**15**	**4.6**	**308**	**2012–2020**		
**ZEA**	100	50	50	816	2019–2020	-	This study
	226	16	7.0	956	2017	2019	[34]
	20	2	10	<50	2012–2014	2018	[32]
**Total**	**346**	**68**	**19.6**	**956**	**2012–2020**		

**Table 7:** Results obtained in Spanish samples of feed for poultry.

Mycotoxin	Total Samples	Positives	Percentage of Positives	Max. Value Found µg/kg	Samples Collection	Year of Publication	Reference
AFB1	100	13	13	6.9	2019–2020	-	This study
	9	1	11	<2	2012–2014	2018	[32]
**Total**	**109**	**14**	**12.8**	**6.9**	**2012–2020**		
AFB2	100	11	11	3.1	2019–2020	-	This study
	9	0	0	<4	2012–2014	2018	[32]
**Total**	**109**	**11**	**10.1**	**3.1**	**2012–2020**		
AFG1	100	7	7	5.6	2019–2020	-	This study
	9	0	0	<4	2012–2014	2018	[32]
**Total**	**109**	**7**	**6.4**	**5.6**	**2012–2020**		
AFG2	100	14	14	3.9	2019–2020	-	This study
	9	0	0	<4	2012–2014	2018	[32]
**Total**	**109**	**14**	**12.8**	**3.9**	**2012–2020**		
DON	100	71	71	755	2019–2020	-	This study
	9	1	11.1	<250	2012–2014	2018	[32]
**Total**	**109**	**72**	**66.1**	**755**	**2012–2020**		
OTA	100	5	5	23.2	2019–2020	-	This study
	9	1	11.1	<25	2012–2014	2018	[32]
**Total**	**109**	**6**	**5.5**	**23.2**	**2012–2020**		
STER	100	7	7	5.1	2019–2020	-	This study
**Total**	**100**	**7**	**7.0**	**5.1**	**2019–2020**		
ZEA	100	66	66	489	2019–2020	-	This study
	9	1	11.1	<50	2012–2014	2018	[32]
**Total**	**109**	**67**	**61.5**	**489**	**2012–2020**		

**Table 8:** Results obtained in Spanish samples of feed for sheep.

Mycotoxin	Total Samples	Positives	Percentage of Positives	Max. Value Found µg/kg	Samples Collection	Year of Publication	Reference
AFB1	100	12	12	6.1	2019–2020	-	This study
	17	1	5.9	<2	2012–2014	2018	[32]
**Total**	**117**	**13**	**11.1**	**6.1**	**2012–2020**		
AFB2	100	15	15	4.9	2019–2020	-	This study
	17	0	0	<4	2012–2014	2018	[32]
**Total**	**117**	**15**	**12.8**	**4.9**	**2012–2020**		
AFG1	100	10	10	6.5	2019–2020	-	This study
	17	0	0	<4	2012–2014	2018	[32]
**Total**	**117**	**10**	**8.5**	**6.5**	**2012–2020**		
AFG2	100	16	16	4	2019–2020	-	This study
	17	0	0	<4	2012–2014	2018	[32]
**Total**	**117**	**16**	**13.7**	**4**	**2012–2020**		
DON	100	72	72	887	2019–2020	-	This study
	17	2	11.8	<250	2012–2014	2018	[32]
**Total**	**117**	**74**	**63.2**	**887**	**2012–2020**		
OTA	100	8	8	45.3	2019–2020	-	This study
	17	5	29.4	<25	2012–2014	2018	[32]
**Total**	**117**	**13**	**11.1**	**45.3**	**2012–2020**		
STER	100	5	5	5.6	2019–2020	-	This study
**Total**	**100**	**5**	**5**	**5.6**	**2012–2020**		
ZEA	100	52	52	658	2019–2020	-	This study
	17	3	17.6	104.4	2012–2014	2018	[32]
**Total**	**117**	**55**	**47.0**	**658**	**2012–2020**		

**Table 9:** Global results obtained in samples of feed from Spain, published in the last 5 years and collected between 2012–2020.

Mycotoxin	Total Samples	Positives (>LOQ)	Percentage of Positives	Max. Value Found (µg/kg)	EU Regulation (µg/kg)	Percentage> EU Regulation	Percentage> EU Lowest Maximum Levels in Southern Europe [16]
AFB1	700	77	11.0	6.9	5–20 [27]	1.1	7.4
AFB2	678	54	8.0	4.9	-		
AFG1	678	36	5.3	6.5	-		
AFG2	678	56	8.3	4.4	-		
DON	678	306	45.1	887	900–5000 [26]	0	11.7
OTA	678	42	6.2	65.5	50–100 [26]	0.15	0.9
STER	626	33	5.3	308	-		
ZEA	678	240	35.4	956	100–500 [26]	2.5	11.8

**Table 10:** Ranges and limits of quantification and detection of mycotoxins in feed.

	AFG2	AFG1	AFB2	AFB1	OTB	ZEA	OTA
Range (µg/kg)	1.26–12.6	4–40	1.26–12.6	4–40	10–100	84–840	10–100
LOQ (µg/kg)	1.26	4	1.26	4	10	84	10
LOD (µg/kg)	0.63	2	0.63	2	5	42	5

LOQ: limit of quantification. LOD: limit of detection.

## Data Availability

The data presented in this study are available in Supplementary Materials.

## Supplementary Materials

The following supporting information can be downloaded at: https://www.mdpi.com/article/10.3390/toxins15030172/s1, Tables S1–S4: Calibration curves. Table S5: Retention times for calibrators and samples for each mycotoxin. Tables S6–S9: Levels of mycotoxins in feed for different species. Table S10: Correlation matrix.

## Abbreviations

ACN	Acetonitrile
AFB1	Aflatoxin B1
AFB2	Aflatoxin B2
AFG1	Aflatoxin G1
AFG2	Aflatoxin G2
AFs	Aflatoxins
ANOVA	Analysis of variance
DON	Deoxynivalenol
EFSA	European Food Safety Authority
ELISA	Enzyme-linked immunosorbent assay
EU	European Union
FB1	Fumonisin B1
FB2	Fumonisin B2
FLD	Fluorescence detector
FUMs	Fumonisins
IARC	International Agency of Research in Cancer
LC	Liquid chromatography
LOD	Limit of detection
LOQ	Limit of quantification
MeOH	Methanol
OTA	Ochratoxin A
OTB	Ochratoxin B
rpm	Revolutions per minute
RSD	Relative standard deviation
STER	Sterigmatocystin
UVE	Photochemical reactor for the derivatization of aflatoxins with UV light
ZEA	Zearalenone
