# Supplementary material for: Co-Occurrence of Mycotoxins in Feed for Cattle, Pigs, Poultry, and Sheep in Navarra, a Region of Northern Spain
Source: Toxins (Basel). 2023 Feb 22;15(3):172. doi: 10.3390/toxins15030172 (PMC10057204; doi:10.3390/toxins15030172)
Supplement: Supplementary file 1 [file toxins-15-00172-s001.zip › toxins-2241128-supplementary.pdf]

Supplementary Materials: Co-Occurrence of Mycotoxins in Feed for Cattle, Pigs, Poultry, and Sheep in Navarra, a Region of Northern Spain

Borja Muñoz-Solano and Elena González-Peñas

Table S1. Obtained calibration curves in the chromatographic sequence in which feed for cattle was analysed.

ng/mL	AFG2		AFB2	
	B.C. (ng/mL)	RE (%)	B.C. (ng/mL)	RE (%)
0.126	0.119	5.5	0.117	7.2
0.189	0.180	4.9	0.181	4.5
0.252	0.252	0.0	0.248	1.5
0.315	0.315	0.1	0.315	0.0
0.504	0.515	2.1	0.521	3.4
0.63	0.645	2.5	0.646	2.6
1.26	1.250	0.8	1.248	1.0
	y=17.82x+0.83	R^2=0.9994	y=57.62x+0.55	R^2=0.9990
ng/mL	**OTA**		**OTB**	
	B.C. (ng/mL)	RE (%)	B.C. (ng/mL)	RE (%)
1	1.1	6.0	1.0	3.2
1.5	1.5	2.2	1.5	3.1
2	2.0	0.9	1.9	3.2
2.5	2.4	4.0	2.6	2.6
4	4.0	0.4	4.0	0.6
5	5.1	1.7	5.0	0.0
10	10.0	0.2	10.0	0.1
	y=7.19x+1.46	R^2=0.9996	y=6.31x+0.61	R^2=0.9998
ng/mL	**AFG1**		**AFB1**	
	B.C. (ng/mL)	RE (%)	B.C. (ng/mL)	RE (%)
0.4	0.4	5.9	0.4	6.0
0.6	0.6	2.9	0.6	0.1
0.8	0.8	0.6	0.8	3.2
1	1.0	1.5	1.0	3.2
1.6	1.6	1.4	1.6	0.3
2	2.1	3.8	2.0	1.9
4	4.0	0.9	4.0	0.3
	y=7.73x+0.18	R^2=0.9991	y=23.68x+0.31	R^2=0.9996
ng/mL	**ZEA**			
	B.C. (ng/mL)	RE (%)	B.C. (ng/mL)	RE (%)
8.4	7.8	7.5		
12.6	12.3	2.6		

16.8	17.0	0.9	
21	21.4	1.7	
33.6	33.0	1.8	
42	43.6	3.8	
84	83.4	0.7	
	y=0.25x+1.74	R^2=0.9990	

Table S2. Obtained calibration curves in the chromatographic sequence in which feed for pigs was analysed.

ng/mL	AFG2		AFB2	
	B.C. (ng/mL)	RE (%)	B.C. (ng/mL)	RE (%)
0.126	0.119	5.9	0.118	6.6
0.189	0.184	2.5	0.183	3.1
0.252	0.249	1.0	0.245	3.0
0.315	0.309	1.8	0.314	0.3
0.504	0.514	2.0	0.521	3.4
0.63	0.652	3.5	0.648	2.8
1.26	1.248	0.9	1.248	1.0
	y=17.58x+1.19	R^2=0.9991	y=57.91x+0.51	R^2=0.9990
ng/mL	**OTA**		**OTB**	
	B.C. (ng/mL)	RE (%)	B.C. (ng/mL)	RE (%)
1	1.1	5.7	0.9	6.3
1.5	1.5	2.1	1.5	2.8
2	2.0	0.2	1.9	3.9
2.5	2.4	3.9	2.6	4.2
4	4.0	0.5	4.1	2.2
5	5.1	2.2	5.0	1.0
10	10.0	0.3	9.9	0.6
	y=7.18x+1.51	R^2=0.9995	y=6.20x+0.81	R^2=0.9994
ng/mL	**AFG1**		**AFB1**	
	B.C. (ng/mL)	RE (%)	B.C. (ng/mL)	RE (%)
0.4	0.4	5.0	0.4	8.0
0.6	0.6	2.6	0.6	1.0
0.8	0.8	3.3	0.8	4.0
1	1.0	0.6	1.0	2.9
1.6	1.6	1.5	1.6	0.2
2	2.1	3.4	2.0	1.2
4	4.0	0.9	4.0	0.1
	y=7.72x+0.09	R^2=0.9991	y=23.72x+0.18	R^2=0.9996
ng/mL	**ZEA**			
	B.C. (ng/mL)	RE (%)	B.C. (ng/mL)	RE (%)
8.4	7.7	8.2		
12.6	12.7	0.4		
16.8	16.8	0.2		

21	20.4	2.8	
33.6	34.0	1.3	
42	43.6	3.8	
84	83.3	0.9	
	y=0.25x+1.70	R^2=0.9990	

Table S3. Obtained calibration curves in the chromatographic sequence in which feed for poultry was analysed.

ng/mL	AFG2		AFB2	
	B.C. (ng/mL)	RE (%)	B.C. (ng/mL)	RE (%)
0.126	0.118	6.1	0.114	9.6
0.189	0.185	1.9	0.178	5.7
0.252	0.246	2.5	0.247	2.0
0.315	0.309	1.8	0.313	0.7
0.504	0.523	3.7	0.527	4.6
0.63	0.646	2.6	0.654	3.9
1.26	1.248	0.9	1.243	1.3
	y=17.50x+1.14	R^2=0.9990	y=57.59x+0.55	R^2=0.9982
ng/mL	OTA		OTB	
	B.C. (ng/mL)	RE (%)	B.C. (ng/mL)	RE (%)
1	1.1	7.2	0.9	5.8
1.5	1.5	3.0	1.4	3.4
2	2.0	1.1	1.9	3.8
2.5	2.4	4.7	2.6	3.7
4	4.0	0.5	4.1	2.3
5	5.1	2.2	5.1	1.2
10	10.0	0.2	9.9	0.6
	y=7.17x+1.50	R^2=0.9994	y=6.19x+0.84	R^2=0.9994
ng/mL	AFG1		AFB1	
	B.C. (ng/mL)	RE (%)	B.C. (ng/mL)	RE (%)
0.4	0.4	8.6	0.4	7.2
0.6	0.6	3.0	0.6	0.5
0.8	0.8	4.2	0.8	4.2
1	1.0	1.5	1.0	3.7
1.6	1.6	2.3	1.6	0.7
2	2.1	4.0	2.0	1.9
4	4.0	1.1	4.0	0.3
	y=7.70x+0.08	R^2=0.9986	y=23.66x+0.32	R^2=0.9995
ng/mL	ZEA			
	B.C. (ng/mL)	RE (%)	B.C. (ng/mL)	RE (%)
8.4	7.6	9.0		
12.6	12.1	4.3		
16.8	16.6	1.3		
21	21.0	0.2		

33.6	34.3	2.1	
42	43.8	4.4	
84	83.0	1.2	
	y=0.24x+1.74	R^2=0.9986	

Table S4. Obtained calibration curves in the chromatographic sequence in which feed for sheep was analysed.

ng/mL	AFG2		AFB2	
	B.C. (ng/mL)	RE (%)	B.C. (ng/mL)	RE (%)
0.126	0.118	6.0	0.118	6.7
0.189	0.175	7.6	0.180	5.0
0.252	0.258	2.4	0.244	3.1
0.315	0.312	1.1	0.311	1.4
0.504	0.525	4.3	0.531	5.3
0.63	0.638	1.2	0.648	2.9
1.26	1.250	0.8	1.245	1.2
	y=17.78x+0.91	R2=0.9990	y=57.60x+0.64	R2=0.9984
ng/mL	**OTA**		**OTB**	
	B.C. (ng/mL)	RE (%)	B.C. (ng/mL)	RE (%)
1	1.1	8.2	0.9	8.4
1.5	1.5	2.1	1.5	3.2
2	2.0	0.7	1.9	3.0
2.5	2.4	5.4	2.6	3.7
4	4.0	0.0	4.1	2.6
5	5.1	2.6	5.1	1.3
10	10.0	0.3	9.9	0.7
	y=7.13x+1.64	R2=0.9992	y=6.21x+0.77	R2=0.9993
ng/mL	**AFG1**		**AFB1**	
	B.C. (ng/mL)	RE (%)	B.C. (ng/mL)	RE (%)
0.4	0.4	2.4	0.4	7.2
0.6	0.6	3.8	0.6	0.5
0.8	0.8	4.3	0.8	4.2
1	1.0	1.6	1.0	3.7
1.6	1.6	1.5	1.6	0.7
2	2.1	2.9	2.0	1.9
4	4.0	0.8	4.0	0.3
	y=7.73x+0.10	R2=0.9992	y=23.66x+0.32	R2=0.9994
ng/mL	**ZEA**			
	B.C. (ng/mL)	RE (%)	B.C. (ng/mL)	RE (%)
8.4	7.5	10.4		
12.6	12.8	1.3		
16.8	16.8	0.2		
21	21.0	0.1		
33.6	34.2	1.7		

42	42.6	1.4	
84	83.5	0.6	
	y=0.25x+1.85	R2=0.9996	

Table S5. Retention times for calibrators and samples for each mycotoxin.

	AFG2	AFG1	AFB2	AFB1	OTB	ZEA	OTA
Calibrators	11.06 ± 0.09	15.28 ± 0.08	19.60 ± 0.10	26.62 ± 0.05	33.58 ± 0.10	37.44 ± 0.09	39.93 ± 0.10
Samples	11.10 ± 0.10	15.30 ± 0.10	19.40 ± 0.10	26.70 ± 0.11	ND	37.50 ± 0,10	39.95 ± 0.09

Table S6. Levels (μg/kg) of mycotoxins in feed for cattle.

Sample	AFG2	AFG1	AFB2	AFB1	OTB	ZEA	OTA	DON	STER
cow-1	<LOD*	<LOD	<LOD	<LOD	<LOD	147	<LOD	<LOQ*	<LOQ
cow-2	<LOD	<LOD	1.37	<LOD	<LOD	239	<LOD	217	<LOQ
cow-3	<LOD	*2.47*	<LOD	<LOD	<LOD	<LOD	<LOD	<LOQ	<LOQ
cow-4	2.51	<LOD	<LOD	<LOD	<LOD	<LOD	*6.91*	321	<LOQ
cow-5	<LOD	<LOD	<LOD	<LOD	<LOD	158	<LOD	115	<LOQ
cow-6	<LOD	<LOD	<LOD	<LOD	<LOD	169	<LOD	278	<LOQ
cow-7	<LOD	<LOD	<LOD	<LOD	<LOD	123	<LOD	402	<LOQ
cow-8	<LOD	<LOD	<LOD	<LOD	<LOD	<LOD	<LOD	<LOQ	<LOQ
cow-9	<LOD	<LOD	<LOD	*3.34*	<LOD	*66*	<LOD	<LOQ	<LOQ
cow-10	<LOD	<LOD	<LOD	<LOD	<LOD	<LOD	<LOD	<LOQ	<LOQ
cow-11	<LOD	<LOD	<LOD	*3.06*	<LOD	*78*	<LOD	123	<LOQ
cow-12	<LOD	<LOD	<LOD	<LOD	<LOD	<LOD	<LOD	<LOQ	<LOQ
cow-13	2.11	<LOD	<LOD	<LOD	<LOD	334	<LOD	297	<LOQ
cow-14	<LOD	<LOD	<LOD	<LOD	<LOD	*58*	<LOD	321	<LOQ
cow-15	<LOD	*2.92*	<LOD	<LOD	<LOD	*50*	<LOD	125	<LOQ
cow-16	<LOD	<LOD	<LOD	<LOD	<LOD	<LOD	<LOD	247	<LOQ
cow-17	<LOD	<LOD	<LOD	<LOD	<LOD	258	<LOD	164	<LOQ
cow-18	<LOD	<LOD	<LOD	<LOD	<LOD	<LOD	*7.74*	230	<LOQ
cow-19	<LOD	<LOD	2.56	<LOD	<LOD	<LOD	<LOD	87	<LOQ
cow-20	<LOD	<LOD	<LOD	<LOD	<LOD	146	<LOD	95	<LOQ
cow-21	<LOD	<LOD	<LOD	<LOD	<LOD	<LOD	<LOD	99	<LOQ
cow-22	<LOD	<LOD	<LOD	4.31	<LOD	*60*	<LOD	97	<LOQ
cow-23	<LOD	<LOD	<LOD	<LOD	<LOD	*68*	<LOD	101	<LOQ
cow-24	*0.66*	<LOD	*0.78*	<LOD	<LOD	*79*	<LOD	<LOQ	<LOQ
cow-25	<LOD	<LOD	<LOD	<LOD	<LOD	<LOD	<LOD	<LOQ	<LOQ
cow-26	<LOD	<LOD	<LOD	<LOD	<LOD	<LOD	<LOD	100	<LOQ
cow-27	<LOD	<LOD	<LOD	<LOD	<LOD	*65*	<LOD	157	<LOQ
cow-28	<LOD	<LOD	1.49	5.43	<LOD	<LOD	<LOD	169	<LOQ
cow-29	<LOD	<LOD	<LOD	<LOD	<LOD	314	<LOD	<LOQ	<LOQ
cow-30	<LOD	<LOD	<LOD	<LOD	<LOD	210	<LOD	214	<LOQ
cow-31	<LOD	<LOD	<LOD	<LOD	<LOD	156	<LOD	201	<LOQ
cow-32	1.97	*3.41*	<LOD	<LOD	<LOD	<LOD	<LOD	364	<LOQ
cow-33	<LOD	<LOD	<LOD	<LOD	<LOD	<LOD	<LOD	287	<LOQ
cow-34	<LOD	<LOD	<LOD	<LOD	<LOD	*69*	<LOD	304	<LOQ
cow-35	<LOD	<LOD	<LOD	<LOD	<LOD	<LOD	<LOD	206	<LOQ
cow-36	<LOD	<LOD	*0.94*	<LOD	<LOD	<LOD	<LOD	127	<LOQ

Sample	AFG2	AFG1	AFB2	AFB1	OTB	ZEA	OTA	DON	STER
cow-37	<LOD	<LOD	<LOD	<LOD	<LOD	90.6	<LOD	156	<LOQ
cow-38	<LOD	<LOD	<LOD	*2.47*	<LOD	<LOD	<LOD	187	<LOQ
cow-39	*0.97*	<LOD	<LOD	<LOD	<LOD	110	<LOD	164	<LOQ
cow-40	<LOD	<LOD	<LOD	<LOD	<LOD	<LOD	<LOD	<LOQ	<LOQ
cow-41	<LOD	<LOD	1.98	<LOD	<LOD	<LOD	<LOD	97	<LOQ
cow-42	<LOD	<LOD	<LOD	<LOD	<LOD	*51*	<LOD	<LOQ	<LOQ
cow-43	<LOD	<LOD	<LOD	<LOD	<LOD	*58*	<LOD	132	<LOQ
cow-44	<LOD	<LOD	<LOD	<LOD	<LOD	*76*	<LOD	125	<LOQ
cow-45	*0.83*	*3.03*	<LOD	<LOD	<LOD	*73*	<LOD	120	<LOQ
cow-46	<LOD	<LOD	<LOD	<LOD	<LOD	<LOD	<LOD	117	<LOQ
cow-47	<LOD	<LOD	<LOD	<LOD	<LOD	<LOD	<LOD	99	<LOQ
cow-48	<LOD	<LOD	<LOD	*2.85*	<LOD	<LOD	<LOD	105	<LOQ
cow-49	<LOD	<LOD	<LOD	<LOD	<LOD	95.7	<LOD	132	<LOQ
cow-50	<LOD	<LOD	<LOD	<LOD	<LOD	<LOD	*5.39*	165	<LOQ
cow-51	<LOD	<LOD	<LOD	<LOD	<LOD	*80*	<LOD	154	<LOQ
cow-52	<LOD	<LOD	<LOD	<LOD	<LOD	<LOD	<LOD	197	<LOQ
cow-53	<LOD	<LOD	<LOD	<LOD	<LOD	85.6	<LOD	146	<LOQ
cow-54	<LOD	<LOD	<LOD	*3.14*	<LOD	<LOD	<LOD	198	<LOQ
cow-55	3.25	<LOD	<LOD	<LOD	<LOD	<LOD	<LOD	177	<LOQ
cow-56	<LOD	<LOD	<LOD	<LOD	<LOD	<LOD	<LOD	161	<LOQ
cow-57	<LOD	<LOD	<LOD	<LOD	<LOD	101	<LOD	147	<LOQ
cow-58	<LOD	<LOD	<LOD	<LOD	<LOD	<LOD	<LOD	<LOQ	<LOQ
cow-59	<LOD	<LOD	<LOD	<LOD	<LOD	<LOD	<LOD	115	<LOQ
cow-60	<LOD	<LOD	<LOD	<LOD	<LOD	99.8	<LOD	136	<LOQ
cow-61	<LOD	<LOD	<LOD	<LOD	<LOD	<LOD	<LOD	107	<LOQ
cow-62	<LOD	<LOD	<LOD	<LOD	<LOD	<LOD	<LOD	<LOQ	<LOQ
cow-63	1.29	<LOD	1.32	<LOD	<LOD	118	<LOD	115	<LOQ
cow-64	<LOD	<LOD	<LOD	<LOD	<LOD	<LOD	<LOD	<LOQ	<LOQ
cow-65	<LOD	<LOD	<LOD	<LOD	<LOD	<LOD	<LOD	<LOQ	<LOQ
cow-66	<LOD	<LOD	<LOD	*2.41*	<LOD	214	*5.87*	189	<LOQ
cow-67	<LOD	<LOD	<LOD	<LOD	<LOD	254	<LOD	117	<LOQ
cow-68	<LOD	<LOD	<LOD	<LOD	<LOD	126	<LOD	235	<LOQ
cow-69	<LOD	<LOD	<LOD	<LOD	<LOD	<LOD	*6.64*	97	<LOQ
cow-70	<LOD	<LOD	<LOD	<LOD	<LOD	<LOD	<LOD	123	<LOQ
cow-71	<LOD	<LOD	<LOD	<LOD	<LOD	<LOD	<LOD	<LOQ	<LOQ
cow-72	<LOD	*2.66*	<LOD	<LOD	<LOD	<LOD	<LOD	<LOQ	<LOQ
cow-73	<LOD	<LOD	<LOD	<LOD	<LOD	98.8	<LOD	165	<LOQ
cow-74	<LOD	<LOD	<LOD	<LOD	<LOD	<LOD	<LOD	<LOQ	<LOQ
cow-75	<LOD	<LOD	<LOD	<LOD	<LOD	88.5	<LOD	177	<LOQ
cow-76	<LOD	<LOD	3.21	4.87	<LOD	<LOD	<LOD	189	<LOQ
cow-77	<LOD	<LOD	<LOD	<LOD	<LOD	114	<LOD	115	<LOQ
cow-78	<LOD	<LOD	<LOD	<LOD	<LOD	<LOD	<LOD	165	<LOQ
cow-79	<LOD	<LOD	<LOD	<LOD	<LOD	155	<LOD	235	<LOQ
cow-80	<LOD	<LOD	<LOD	<LOD	<LOD	<LOD	<LOD	81	<LOQ
cow-81	<LOD	<LOD	*0.7*	<LOD	<LOD	<LOD	<LOD	<LOQ	3.3
cow-82	<LOD	<LOD	<LOD	<LOD	<LOD	<LOD	<LOD	101	3.9

Sample	AFG2	AFG1	AFB2	AFB1	OTB	ZEA	OTA	DON	STER
cow-83	<LOD	<LOD	<LOD	<LOD	<LOD	<LOD	<LOD	<LOQ	<LOQ
cow-84	2.1	*2.87*	<LOD	<LOD	<LOD	413	<LOD	574	<LOQ
cow-85	<LOD	<LOD	<LOD	*2.76*	<LOD	280	<LOD	434	2.6
cow-86	<LOD	<LOD	*0.76*	<LOD	<LOD	<LOD	<LOD	<LOQ	<LOQ
cow-87	<LOD	<LOD	<LOD	<LOD	<LOD	<LOD	<LOD	123	<LOQ
cow-88	<LOD	<LOD	<LOD	<LOD	<LOD	147	<LOD	257	<LOQ
cow-89	<LOD	<LOD	<LOD	<LOD	<LOD	<LOD	<LOD	159	<LOQ
cow-90	<LOD	<LOD	<LOD	*2.13*	<LOD	*69*	<LOD	192	<LOQ
cow-91	<LOD	<LOD	<LOD	<LOD	<LOD	*73*	<LOD	<LOQ	<LOQ
cow-92	<LOD	<LOD	<LOD	<LOD	<LOD	*81*	<LOD	115	<LOQ
cow-93	<LOD	<LOD	<LOD	<LOD	<LOD	<LOD	<LOD	87	<LOQ
cow-94	<LOD	<LOD	<LOD	<LOD	<LOD	<LOD	<LOD	74	<LOQ
cow-95	<LOD	*3.12*	<LOD	<LOD	<LOD	273	<LOD	125	<LOQ
cow-96	<LOD	<LOD	<LOD	<LOD	<LOD	98.7	<LOD	196	4.7
cow-97	<LOD	<LOD	<LOD	<LOD	<LOD	<LOD	<LOD	108	3.1
cow-98	<LOD	<LOD	<LOD	<LOD	<LOD	*79*	*4.98*	<LOQ	<LOQ
cow-99	<LOD	<LOD	<LOD	<LOD	<LOD	<LOD	<LOD	<LOQ	<LOQ
cow-100	<LOD	<LOD	*0.89*	4.12	<LOD	85.9	<LOD	376	2.2
*LOD (ug/kg)	0.63	2.0	0.63	2.0	5.0	42.0	5.0		
*LOQ (ug/kg)	1.26	4.0	1.26	4.0	10.0	84.0	10.0	74.0	0.8

Italics: value >LOD <LOQ

Table S7. Levels (μg/kg) of mycotoxins in feed for pigs.

Sample	AFG2	AFG1	AFB2	AFB1	OTB	ZEA	OTA	DON	STER
pig-1	<LOD*	<LOD	<LOD	<LOD	<LOD	106	<LOD	87	<LOQ*
pig-2	2.52	<LOD	<LOD	<LOD	<LOD	87.9	<LOD	121	<LOQ
pig-3	<LOD	<LOD	<LOD	<LOD	<LOD	<LOD	*7.24*	<LOQ	<LOQ
pig-4	<LOD	<LOD	2.78	5.12	<LOD	<LOD	<LOD	221	<LOQ
pig-5	<LOD	<LOD	<LOD	<LOD	<LOD	<LOD	<LOD	157	<LOQ
pig-6	2.98	<LOD	<LOD	<LOD	<LOD	122	<LOD	198	<LOQ
pig-7	<LOD	<LOD	<LOD	<LOD	<LOD	<LOD	<LOD	231	<LOQ
pig-8	<LOD	<LOD	<LOD	<LOD	<LOD	369	*8.36*	89	<LOQ
pig-9	<LOD	<LOD	<LOD	<LOD	<LOD	<LOD	<LOD	96	<LOQ
pig-10	<LOD	<LOD	<LOD	<LOD	<LOD	*76*	<LOD	101	<LOQ
pig-11	*0.99*	<LOD	<LOD	<LOD	<LOD	*81*	<LOD	331	<LOQ
pig-12	<LOD	<LOD	<LOD	<LOD	<LOD	<LOD	<LOD	233	<LOQ
pig-13	<LOD	4.85	3.12	<LOD	<LOD	816	<LOD	410	<LOQ
pig-14	<LOD	<LOD	<LOD	<LOD	<LOD	<LOD	<LOD	<LOQ	<LOQ
pig-15	<LOD	<LOD	<LOD	<LOD	<LOD	<LOD	<LOD	225	<LOQ
pig-16	4.35	<LOD	*0.74*	<LOD	<LOD	668	<LOD	121	<LOQ
pig-17	<LOD	<LOD	<LOD	<LOD	<LOD	<LOD	<LOD	<LOQ	<LOQ
pig-18	<LOD	<LOD	<LOD	<LOD	<LOD	223	<LOD	301	<LOQ
pig-19	*0.67*	<LOD	<LOD	<LOD	<LOD	*66*	<LOD	<LOQ	<LOQ
pig-20	<LOD	<LOD	<LOD	<LOD	<LOD	*68*	<LOD	<LOQ	<LOQ
pig-21	<LOD	<LOD	1.36	<LOD	<LOD	*71*	<LOD	225	<LOQ
pig-22	<LOD	<LOD	<LOD	<LOD	<LOD	<LOD	<LOD	87	<LOQ

Sample	AFG2	AFG1	AFB2	AFB1	OTB	ZEA	OTA	DON	STER
pig-23	<LOD	<LOD	<LOD	6.13	<LOD	328	<LOD	99	<LOQ
pig-24	*0.76*	<LOD	<LOD	<LOD	<LOD	<LOD	<LOD	84	<LOQ
pig-25	<LOD	<LOD	<LOD	<LOD	<LOD	<LOD	<LOD	76	<LOQ
pig-26	<LOD	<LOD	<LOD	<LOD	<LOD	<LOD	<LOD	102	2.1
pig-27	<LOD	<LOD	<LOD	<LOD	<LOD	236	<LOD	281	<LOQ
pig-28	<LOD	<LOD	<LOD	<LOD	<LOD	<LOD	<LOD	191	<LOQ
pig-29	<LOD	<LOD	<LOD	<LOD	<LOD	<LOD	<LOD	193	3.9
pig-30	<LOD	<LOD	1.89	<LOD	<LOD	<LOD	<LOD	<LOQ	<LOQ
pig-31	*0.7*	<LOD	1.97	5.89	<LOD	114	<LOD	111	<LOQ
pig-32	<LOD	<LOD	<LOD	<LOD	<LOD	<LOD	<LOD	<LOQ	<LOQ
pig-33	<LOD	<LOD	<LOD	<LOD	<LOD	<LOD	<LOD	89	<LOQ
pig-34	<LOD	<LOD	<LOD	<LOD	<LOD	<LOD	<LOD	187	<LOQ
pig-35	<LOD	<LOD	<LOD	<LOD	<LOD	89.9	<LOD	145	<LOQ
pig-36	<LOD	<LOD	<LOD	<LOD	<LOD	98.5	65.5	162	<LOQ
pig-37	<LOD	<LOD	<LOD	<LOD	<LOD	107	<LOD	87	<LOQ
pig-38	<LOD	<LOD	<LOD	<LOD	<LOD	<LOD	<LOD	130	<LOQ
pig-39	<LOD	<LOD	<LOD	<LOD	<LOD	<LOD	<LOD	145	<LOQ
pig-40	3.12	5.1	<LOD	<LOD	<LOD	<LOD	<LOD	117	<LOQ
pig-41	<LOD	<LOD	<LOD	<LOD	<LOD	<LOD	<LOD	89	<LOQ
pig-42	<LOD	<LOD	<LOD	<LOD	<LOD	<LOD	<LOD	96	<LOQ
pig-43	3.98	*3.08*	<LOD	<LOD	<LOD	389	<LOD	187	<LOQ
pig-44	<LOD	<LOD	2.25	4.63	<LOD	92.3	*5.91*	130	<LOQ
pig-45	<LOD	<LOD	<LOD	<LOD	<LOD	<LOD	<LOD	<LOQ	<LOQ
pig-46	<LOD	<LOD	<LOD	<LOD	<LOD	<LOD	<LOD	<LOQ	<LOQ
pig-47	<LOD	<LOD	<LOD	<LOD	<LOD	105	<LOD	<LOQ	<LOQ
pig-48	<LOD	<LOD	<LOD	<LOD	<LOD	<LOD	<LOD	145	<LOQ
pig-49	<LOD	6.03	<LOD	6.24	<LOD	<LOD	<LOD	<LOQ	<LOQ
pig-50	<LOD	<LOD	*0.81*	<LOD	<LOD	<LOD	<LOD	<LOQ	<LOQ
pig-51	<LOD	<LOD	<LOD	<LOD	<LOD	<LOD	<LOD	165	<LOQ
pig-52	<LOD	<LOD	<LOD	<LOD	<LOD	168	<LOD	314	<LOQ
pig-53	<LOD	<LOD	<LOD	<LOD	<LOD	<LOD	<LOD	<LOQ	<LOQ
pig-54	<LOD	<LOD	<LOD	<LOD	<LOD	142	<LOD	225	<LOQ
pig-55	1.31	*2.64*	<LOD	<LOD	<LOD	115	<LOD	76	<LOQ
pig-56	<LOD	<LOD	<LOD	<LOD	<LOD	<LOD	<LOD	<LOQ	<LOQ
pig-57	<LOD	<LOD	<LOD	<LOD	<LOD	<LOD	<LOD	<LOQ	<LOQ
pig-58	<LOD	<LOD	<LOD	<LOD	<LOD	160	<LOD	158	<LOQ
pig-59	<LOD	<LOD	2.87	<LOD	<LOD	<LOD	<LOD	112	<LOQ
pig-60	<LOD	<LOD	<LOD	<LOD	<LOD	*74*	<LOD	<LOQ	<LOQ
pig-61	*0.84*	<LOD	<LOD	<LOD	<LOD	*80*	<LOD	223	<LOQ
pig-62	<LOD	<LOD	<LOD	<LOD	<LOD	*59*	<LOD	<LOQ	<LOQ
pig-63	<LOD	<LOD	<LOD	<LOD	<LOD	*74*	<LOD	278	<LOQ
pig-64	<LOD	<LOD	<LOD	<LOD	<LOD	<LOD	<LOD	91	<LOQ
pig-65	<LOD	*2.15*	<LOD	<LOD	<LOD	<LOD	<LOD	100	<LOQ
pig-66	<LOD	<LOD	<LOD	<LOD	<LOD	<LOD	<LOD	<LOQ	<LOQ
pig-67	<LOD	<LOD	<LOD	<LOD	<LOD	254	42.3	129	<LOQ
pig-68	<LOD	4.51	<LOD	<LOD	<LOD	<LOD	<LOD	100	<LOQ

Sample	AFG2	AFG1	AFB2	AFB1	OTB	ZEA	OTA	DON	STER
pig-69	<LOD	<LOD	<LOD	<LOD	<LOD	<LOD	<LOD	101	<LOQ
pig-70	1.59	<LOD	1.58	<LOD	<LOD	*66*	<LOD	280	<LOQ
pig-71	<LOD	<LOD	<LOD	<LOD	<LOD	*78*	<LOD	148	<LOQ
pig-72	<LOD	<LOD	<LOD	<LOD	<LOD	<LOD	<LOD	169	<LOQ
pig-73	<LOD	<LOD	<LOD	<LOD	<LOD	*62*	<LOD	85	<LOQ
pig-74	<LOD	<LOD	<LOD	<LOD	<LOD	187	<LOD	133	<LOQ
pig-75	<LOD	<LOD	<LOD	<LOD	<LOD	<LOD	<LOD	<LOQ	<LOQ
pig-76	<LOD	<LOD	<LOD	<LOD	<LOD	152	<LOD	124	<LOQ
pig-77	<LOD	<LOD	<LOD	<LOD	<LOD	<LOD	<LOD	<LOQ	<LOQ
pig-78	<LOD	<LOD	<LOD	<LOD	<LOD	*54*	<LOD	101	<LOQ
pig-79	<LOD	<LOD	<LOD	4.87	<LOD	*56*	<LOD	78	<LOQ
pig-80	2.12	<LOD	2.3	<LOD	<LOD	*74*	<LOD	154	<LOQ
pig-81	<LOD	<LOD	<LOD	<LOD	<LOD	<LOD	<LOD	132	2.3
pig-82	<LOD	<LOD	<LOD	<LOD	<LOD	85.3	<LOD	159	<LOQ
pig-83	<LOD	5.32	<LOD	<LOD	<LOD	<LOD	<LOD	147	<LOQ
pig-84	<LOD	<LOD	<LOD	<LOD	<LOD	*81*	<LOD	76	<LOQ
pig-85	<LOD	<LOD	<LOD	<LOD	<LOD	*76*	<LOD	101	2.9
pig-86	1.28	<LOD	3.85	<LOD	<LOD	*79*	<LOD	201	<LOQ
pig-87	1.52	<LOD	<LOD	<LOD	<LOD	<LOD	<LOD	<LOQ	<LOQ
pig-88	<LOD	<LOD	<LOD	<LOD	<LOD	<LOD	14.8	88	3.8
pig-89	<LOD	<LOD	<LOD	<LOD	<LOD	514	<LOD	145	2.6
pig-90	<LOD	<LOD	<LOD	<LOD	<LOD	<LOD	<LOD	<LOQ	<LOQ
pig-91	<LOD	4.66	*0.93*	<LOD	<LOD	*74*	<LOD	346	5.8
pig-92	*0.66*	<LOD	<LOD	<LOD	<LOD	<LOD	<LOD	218	<LOQ
pig-93	<LOD	<LOD	<LOD	<LOD	<LOD	*70*	<LOD	<LOQ	6.1
pig-94	<LOD	<LOD	2.43	<LOD	<LOD	*57*	<LOD	<LOQ	2.5
pig-95	<LOD	<LOD	<LOD	<LOD	<LOD	*63*	<LOD	<LOQ	<LOQ
pig-96	<LOD	<LOD	<LOD	<LOD	<LOD	*80*	15.9	118	<LOQ
pig-97	<LOD	<LOD	<LOD	<LOD	<LOD	<LOD	<LOD	<LOQ	<LOQ
pig-98	<LOD	<LOD	<LOD	<LOD	<LOD	429	<LOD	185	<LOQ
pig-99	2.57	*2.98*	<LOD	<LOD	<LOD	339	<LOD	<LOQ	<LOQ
pig-100	<LOD	<LOD	<LOD	4.25	<LOD	<LOD	<LOD	<LOQ	3.6
*LOD (ug/kg)	0.63	2.0	0.63	2.0	5.0	42.0	5.0		
*LOQ (ug/kg)	1.26	4.0	1.26	4.0	10.0	84.0	10.0	74.0	0.8

Italics: value >LOD <LOQ

Table S8. Levels (µg/kg) of mycotoxins in feed for poultry.

Sample	AFG2	AFG1	AFB2	AFB1	OTB	ZEA	OTA	DON	STER
poultry-1	<LOD*	<LOD	<LOD	*2.1*	<LOD	<LOD	<LOD	268	<LOQ*
poultry-2	<LOD	<LOD	*0.65*	<LOD	<LOD	96	<LOD	127	<LOQ
poultry-3	3.38	4.12	<LOD	<LOD	<LOD	<LOD	<LOD	97	<LOQ
poultry-4	<LOD	<LOD	<LOD	<LOD	<LOD	*76*	<LOD	<LOQ	<LOQ
poultry-5	<LOD	<LOD	*0.71*	5.31	<LOD	258	<LOD	113	<LOQ
poultry-6	*0.73*	<LOD	<LOD	<LOD	<LOD	<LOD	<LOD	214	<LOQ
poultry-7	<LOD	<LOD	<LOD	<LOD	<LOD	212	<LOD	185	<LOQ
poultry-8	<LOD	<LOD	<LOD	<LOD	<LOD	<LOD	<LOD	<LOQ	<LOQ

**Sample**	**AFG2**	**AFG1**	**AFB2**	**AFB1**	**OTB**	**ZEA**	**OTA**	**DON**	**STER**
poultry-9	<LOD	<LOD	<LOD	<LOD	<LOD	<LOD	<LOD	<LOQ	<LOQ
poultry-10	<LOD	<LOD	<LOD	<LOD	<LOD	215	<LOD	115	<LOQ
poultry-11	<LOD	<LOD	<LOD	<LOD	<LOD	124	<LOD	199	<LOQ
poultry-12	<LOD	<LOD	<LOD	<LOD	<LOD	211	<LOD	187	<LOQ
poultry-13	<LOD	5.62	<LOD	<LOD	<LOD	158	14.1	178	<LOQ
poultry-14	<LOD	<LOD	<LOD	<LOD	<LOD	*71*	<LOD	<LOQ	<LOQ
poultry-15	<LOD	<LOD	2.23	4.89	<LOD	<LOD	<LOD	87	<LOQ
poultry-16	<LOD	<LOD	<LOD	<LOD	<LOD	*68*	<LOD	102	<LOQ
poultry-17	<LOD	<LOD	<LOD	<LOD	<LOD	*79*	<LOD	178	<LOQ
poultry-18	<LOD	<LOD	<LOD	<LOD	<LOD	*81*	<LOD	234	<LOQ
poultry-19	<LOD	<LOD	<LOD	<LOD	<LOD	<LOD	<LOD	99	<LOQ
poultry-20	*0.68*	<LOD	<LOD	<LOD	<LOD	152	<LOD	178	<LOQ
poultry-21	<LOD	<LOD	<LOD	5.22	<LOD	97	<LOD	321	<LOQ
poultry-22	<LOD	<LOD	<LOD	<LOD	<LOD	<LOD	<LOD	<LOQ	<LOQ
poultry-23	<LOD	<LOD	<LOD	<LOD	<LOD	<LOD	<LOD	81	<LOQ
poultry-24	2.15	<LOD	<LOD	<LOD	<LOD	298	<LOD	301	<LOQ
poultry-25	<LOD	<LOD	<LOD	<LOD	<LOD	401	<LOD	215	<LOQ
poultry-26	*0.81*	<LOD	<LOD	<LOD	<LOD	<LOD	<LOD	121	<LOQ
poultry-27	<LOD	<LOD	1.32	<LOD	<LOD	110	<LOD	<LOQ	<LOQ
poultry-28	<LOD	<LOD	<LOD	6.11	<LOD	<LOD	<LOD	115	<LOQ
poultry-29	<LOD	<LOD	<LOD	<LOD	<LOD	255	<LOD	231	<LOQ
poultry-30	<LOD	<LOD	<LOD	<LOD	<LOD	<LOD	<LOD	187	<LOQ
poultry-31	<LOD	<LOD	<LOD	<LOD	<LOD	142	<LOD	<LOQ	<LOQ
poultry-32	<LOD	<LOD	<LOD	<LOD	<LOD	241	<LOD	<LOQ	<LOQ
poultry-33	<LOD	<LOD	<LOD	<LOD	<LOD	98.6	<LOD	420	<LOQ
poultry-34	2.63	<LOD	<LOD	<LOD	<LOD	111	<LOD	<LOQ	<LOQ
poultry-35	<LOD	<LOD	<LOD	<LOD	<LOD	<LOD	<LOD	<LOQ	<LOQ
poultry-36	<LOD	<LOD	<LOD	<LOD	<LOD	158	<LOD	332	<LOQ
poultry-37	<LOD	<LOD	2.56	<LOD	<LOD	<LOD	<LOD	212	<LOQ
poultry-38	<LOD	<LOD	<LOD	5.54	<LOD	114	<LOD	225	<LOQ
poultry-39	<LOD	<LOD	<LOD	<LOD	<LOD	389	<LOD	147	<LOQ
poultry-40	2.57	*2.14*	<LOD	<LOD	<LOD	90.5	<LOD	230	<LOQ
poultry-41	<LOD	<LOD	<LOD	<LOD	<LOD	112	<LOD	145	<LOQ
poultry-42	<LOD	<LOD	<LOD	<LOD	<LOD	*78*	<LOD	215	<LOQ
poultry-43	<LOD	<LOD	<LOD	<LOD	<LOD	*82*	<LOD	320	<LOQ
poultry-44	3.9	4.86	<LOD	4.23	<LOD	<LOD	<LOD	<LOQ	<LOQ
poultry-45	<LOD	<LOD	*0.98*	<LOD	<LOD	255	19.6	111	<LOQ
poultry-46	<LOD	<LOD	<LOD	<LOD	<LOD	*54*	<LOD	97	<LOQ
poultry-47	<LOD	<LOD	<LOD	<LOD	<LOD	<LOD	<LOD	<LOQ	2.6
poultry-48	<LOD	<LOD	<LOD	<LOD	<LOD	*61*	<LOD	122	<LOQ
poultry-49	<LOD	<LOD	<LOD	<LOD	<LOD	<LOD	<LOD	<LOQ	<LOQ
poultry-50	*0.94*	5.32	<LOD	<LOD	<LOD	197	<LOD	332	<LOQ
poultry-51	<LOD	<LOD	<LOD	<LOD	<LOD	<LOD	<LOD	<LOQ	<LOQ
poultry-52	<LOD	<LOD	2.21	<LOD	<LOD	*63*	23.2	<LOQ	<LOQ
poultry-53	<LOD	<LOD	<LOD	<LOD	<LOD	*70*	<LOD	452	<LOQ
poultry-54	<LOD	<LOD	<LOD	<LOD	<LOD	<LOD	<LOD	<LOQ	<LOQ

**Sample**	**AFG2**	**AFG1**	**AFB2**	**AFB1**	**OTB**	**ZEA**	**OTA**	**DON**	**STER**
poultry-55	<LOD	<LOD	<LOD	<LOD	<LOD	245	<LOD	325	<LOQ
poultry-56	<LOD	<LOD	<LOD	<LOD	<LOD	<LOD	<LOD	<LOQ	<LOQ
poultry-57	<LOD	<LOD	<LOD	4.78	<LOD	163	<LOD	755	<LOQ
poultry-58	<LOD	<LOD	1.97	<LOD	<LOD	125	<LOD	542	<LOQ
poultry-59	<LOD	<LOD	<LOD	<LOD	<LOD	248	<LOD	400	<LOQ
poultry-60	<LOD	<LOD	<LOD	<LOD	<LOD	*73*	<LOD	<LOQ	<LOQ
poultry-61	<LOD	<LOD	<LOD	*2.21*	<LOD	*49*	<LOD	<LOQ	<LOQ
poultry-62	<LOD	<LOD	<LOD	<LOD	<LOD	*58*	<LOD	412	<LOQ
poultry-63	<LOD	<LOD	<LOD	<LOD	<LOD	<LOD	<LOD	<LOQ	<LOQ
poultry-64	3.11	4.11	<LOD	<LOD	<LOD	122	<LOD	<LOQ	<LOQ
poultry-65	<LOD	<LOD	<LOD	<LOD	<LOD	158	<LOD	222	<LOQ
poultry-66	<LOD	<LOD	<LOD	<LOD	<LOD	431	<LOD	547	<LOQ
poultry-67	<LOD	<LOD	<LOD	<LOD	<LOD	<LOD	<LOD	321	<LOQ
poultry-68	<LOD	<LOD	1.89	<LOD	<LOD	*64*	<LOD	<LOQ	<LOQ
poultry-69	<LOD	<LOD	<LOD	<LOD	<LOD	*56*	<LOD	431	<LOQ
poultry-70	*0.79*	<LOD	<LOD	6.87	<LOD	*81*	<LOD	279	<LOQ
poultry-71	<LOD	<LOD	<LOD	<LOD	<LOD	<LOD	<LOD	335	<LOQ
poultry-72	<LOD	<LOD	<LOD	<LOD	<LOD	321	<LOD	185	<LOQ
poultry-73	<LOD	<LOD	<LOD	<LOD	<LOD	257	<LOD	199	<LOQ
poultry-74	<LOD	<LOD	<LOD	<LOD	<LOD	<LOD	<LOD	257	<LOQ
poultry-75	<LOD	<LOD	<LOD	<LOD	<LOD	<LOD	<LOD	<LOQ	<LOQ
poultry-76	2.59	<LOD	<LOD	<LOD	<LOD	*49*	<LOD	445	<LOQ
poultry-77	<LOD	<LOD	<LOD	<LOD	<LOD	*58*	<LOD	123	<LOQ
poultry-78	<LOD	<LOD	<LOD	<LOD	<LOD	158	<LOD	<LOQ	<LOQ
poultry-79	<LOD	4.26	1.39	<LOD	<LOD	*67*	<LOD	447	<LOQ
poultry-80	<LOD	<LOD	<LOD	<LOD	<LOD	<LOD	<LOD	450	<LOQ
poultry-81	<LOD	<LOD	<LOD	<LOD	<LOD	<LOD	<LOD	125	3.7
poultry-82	<LOD	<LOD	<LOD	<LOD	<LOD	265	<LOD	187	<LOQ
poultry-83	<LOD	<LOD	<LOD	<LOD	<LOD	<LOD	<LOD	196	<LOQ
poultry-84	2.12	<LOD	<LOD	5.21	<LOD	<LOD	<LOD	<LOQ	<LOQ
poultry-85	<LOD	<LOD	<LOD	<LOD	<LOD	489	15.6	332	3.1
poultry-86	<LOD	<LOD	<LOD	<LOD	<LOD	*58*	<LOD	<LOQ	<LOQ
poultry-87	<LOD	<LOD	<LOD	<LOD	<LOD	*69*	<LOD	445	<LOQ
poultry-88	<LOD	<LOD	<LOD	<LOD	<LOD	324	<LOD	221	<LOQ
poultry-89	<LOD	<LOD	<LOD	<LOD	<LOD	<LOD	<LOD	<LOQ	<LOQ
poultry-90	1.51	<LOD	3.12	<LOD	<LOD	141	<LOD	185	2.8
poultry-91	<LOD	<LOD	<LOD	<LOD	<LOD	<LOD	<LOD	198	<LOQ
poultry-92	<LOD	<LOD	<LOD	<LOD	<LOD	*80*	<LOD	87	<LOQ
poultry-93	<LOD	<LOD	<LOD	5.54	<LOD	128	<LOD	321	<LOQ
poultry-94	<LOD	<LOD	<LOD	<LOD	<LOD	<LOD	<LOD	207	5.1
poultry-95	<LOD	<LOD	<LOD	<LOD	<LOD	*87.9*	18.1	378	2.3
poultry-96	<LOD	<LOD	<LOD	6.78	<LOD	*52*	<LOD	247	<LOQ
poultry-97	<LOD	<LOD	<LOD	<LOD	<LOD	<LOD	<LOD	<LOQ	<LOQ
poultry-98	<LOD	<LOD	<LOD	<LOD	<LOD	*49*	<LOD	115	<LOQ
poultry-99	<LOD	<LOD	<LOD	<LOD	<LOD	96.4	<LOD	<LOQ	4.4
poultry-100	<LOD	<LOD	<LOD	<LOD	<LOD	<LOD	<LOD	189	<LOQ

*LOD (ug/kg)	0.63	2.0	0.63	2.0	5.0	42.0	5.0		
*LOQ (ug/kg)	1.26	4.0	1.26	4.0	10.0	84.0	10.0	74.0	0.8

Italics: value >LOD <LOQ

Table S9. Levels (μg/kg) of mycotoxins in feed for sheep.

Sample	AFG2	AFG1	AFB2	AFB1	OTB	ZEA	OTA	DON	STER
sheep-1	<LOD*	<LOD	<LOD	<LOD	<LOD	74	<LOD	<LOQ*	<LOQ
sheep-2	<LOD	<LOD	<LOD	5.13	<LOD	121	<LOD	315	<LOQ
sheep-3	<LOD	<LOD	0.78	<LOD	<LOD	<LOD	<LOD	287	<LOQ
sheep-4	0.64	<LOD	<LOD	<LOD	<LOD	<LOD	<LOD	<LOQ	<LOQ
sheep-5	<LOD	<LOD	<LOD	<LOD	<LOD	60	<LOD	<LOQ	<LOQ
sheep-6	<LOD	<LOD	<LOD	<LOD	<LOD	157	<LOD	97	<LOQ
sheep-7	<LOD	<LOD	<LOD	3.12	<LOD	<LOD	5.47	<LOQ	<LOQ
sheep-8	2.21	<LOD	2.58	<LOD	<LOD	71	<LOD	80	<LOQ
sheep-9	<LOD	<LOD	<LOD	<LOD	<LOD	225	<LOD	122	<LOQ
sheep-10	<LOD	<LOD	<LOD	<LOD	<LOD	<LOD	<LOD	<LOQ	<LOQ
sheep-11	<LOD	5.21	<LOD	<LOD	<LOD	<LOD	<LOD	101	<LOQ
sheep-12	<LOD	<LOD	<LOD	<LOD	<LOD	325	<LOD	354	<LOQ
sheep-13	<LOD	<LOD	<LOD	<LOD	<LOD	<LOD	<LOD	<LOQ	<LOQ
sheep-14	<LOD	<LOD	<LOD	<LOD	<LOD	68	<LOD	200	<LOQ
sheep-15	<LOD	<LOD	3.21	<LOD	<LOD	452	<LOD	258	<LOQ
sheep-16	<LOD	<LOD	<LOD	2.18	<LOD	79	<LOD	<LOQ	<LOQ
sheep-17	<LOD	3.12	<LOD	<LOD	<LOD	<LOD	<LOD	<LOQ	<LOQ
sheep-18	<LOD	<LOD	<LOD	<LOD	<LOD	129	<LOD	113	<LOQ
sheep-19	<LOD	<LOD	<LOD	<LOD	<LOD	<LOD	<LOD	<LOQ	<LOQ
sheep-20	<LOD	<LOD	<LOD	<LOD	<LOD	<LOD	<LOD	136	<LOQ
sheep-21	<LOD	<LOD	4.85	<LOD	<LOD	88.4	15.1	118	<LOQ
sheep-22	3.12	<LOD	<LOD	<LOD	<LOD	125	<LOD	225	<LOQ
sheep-23	<LOD	<LOD	<LOD	<LOD	<LOD	<LOD	<LOD	<LOQ	<LOQ
sheep-24	<LOD	6.54	1.56	6.13	<LOD	452	<LOD	290	<LOQ
sheep-25	<LOD	<LOD	<LOD	<LOD	<LOD	<LOD	<LOD	<LOQ	<LOQ
sheep-26	<LOD	<LOD	<LOD	<LOD	<LOD	46	<LOD	222	<LOQ
sheep-27	2.54	<LOD	<LOD	<LOD	<LOD	57	<LOD	335	<LOQ
sheep-28	2.68	<LOD	<LOD	<LOD	<LOD	<LOD	<LOD	89	<LOQ
sheep-29	<LOD	<LOD	0.93	<LOD	<LOD	226	29.3	101	<LOQ
sheep-30	<LOD	<LOD	<LOD	<LOD	<LOD	<LOD	<LOD	190	<LOQ
sheep-31	<LOD	<LOD	<LOD	<LOD	<LOD	<LOD	<LOD	132	<LOQ
sheep-32	<LOD	<LOD	<LOD	<LOD	<LOD	114	<LOD	154	<LOQ
sheep-33	<LOD	<LOD	<LOD	<LOD	<LOD	<LOD	<LOD	168	5.6
sheep-34	0.79	4.18	<LOD	<LOD	<LOD	<LOD	<LOD	330	<LOQ
sheep-35	<LOD	<LOD	<LOD	3.19	<LOD	97.5	11.2	<LOQ	<LOQ
sheep-36	<LOD	<LOD	1.57	<LOD	<LOD	<LOD	<LOD	115	<LOQ
sheep-37	<LOD	<LOD	<LOD	<LOD	<LOD	165	<LOD	440	<LOQ
sheep-38	<LOD	<LOD	<LOD	<LOD	<LOD	158	<LOD	397	<LOQ
sheep-39	<LOD	<LOD	<LOD	<LOD	<LOD	178	<LOD	232	<LOQ
sheep-40	<LOD	<LOD	<LOD	<LOD	<LOD	<LOD	<LOD	93	<LOQ
sheep-41	<LOD	<LOD	<LOD	<LOD	<LOD	215	<LOD	114	<LOQ

Sample	AFG2	AFG1	AFB2	AFB1	OTB	ZEA	OTA	DON	STER
sheep-42	<LOD	2.87	<LOD	4.21	<LOD	425	<LOD	354	<LOQ
sheep-43	0.87	<LOD	<LOD	<LOD	<LOD	<LOD	45.3	85	<LOQ
sheep-44	<LOD	<LOD	2.1	<LOD	<LOD	501	<LOD	321	<LOQ
sheep-45	<LOD	<LOD	<LOD	<LOD	<LOD	<LOD	<LOD	<LOQ	<LOQ
sheep-46	<LOD	<LOD	<LOD	<LOD	<LOD	458	<LOD	887	<LOQ
sheep-47	<LOD	<LOD	<LOD	<LOD	<LOD	<LOD	<LOD	332	<LOQ
sheep-48	<LOD	4.87	<LOD	<LOD	<LOD	232	<LOD	546	<LOQ
sheep-49	<LOD	<LOD	<LOD	<LOD	<LOD	220	26.8	128	<LOQ
sheep-50	<LOD	<LOD	<LOD	<LOD	<LOD	<LOD	<LOD	741	<LOQ
sheep-51	<LOD	<LOD	<LOD	<LOD	<LOD	<LOD	<LOD	332	<LOQ
sheep-52	<LOD	<LOD	<LOD	<LOD	<LOD	<LOD	<LOD	<LOQ	<LOQ
sheep-53	<LOD	<LOD	<LOD	<LOD	<LOD	338	<LOD	<LOQ	<LOQ
sheep-54	3.58	<LOD	<LOD	<LOD	<LOD	187	<LOD	215	<LOQ
sheep-55	<LOD	<LOD	0.86	<LOD	<LOD	<LOD	<LOD	389	<LOQ
sheep-56	<LOD	<LOD	<LOD	<LOD	<LOD	<LOD	<LOD	466	<LOQ
sheep-57	<LOD	<LOD	<LOD	<LOD	<LOD	<LOD	<LOD	336	<LOQ
sheep-58	<LOD	<LOD	<LOD	4.56	<LOD	248	<LOD	213	<LOQ
sheep-59	3.12	<LOD	<LOD	<LOD	<LOD	68	<LOD	213	<LOQ
sheep-60	<LOD	<LOD	<LOD	<LOD	<LOD	54	<LOD	201	<LOQ
sheep-61	<LOD	<LOD	<LOD	<LOD	<LOD	<LOD	<LOD	302	<LOQ
sheep-62	<LOD	<LOD	<LOD	<LOD	<LOD	<LOD	<LOD	300	<LOQ
sheep-63	<LOD	2.17	<LOD	<LOD	<LOD	658	<LOD	<LOQ	<LOQ
sheep-64	<LOD	<LOD	<LOD	<LOD	<LOD	<LOD	<LOD	<LOQ	<LOQ
sheep-65	<LOD	<LOD	<LOD	<LOD	<LOD	420	<LOD	125	<LOQ
sheep-66	0.68	<LOD	<LOD	<LOD	<LOD	<LOD	<LOD	187	<LOQ
sheep-67	<LOD	<LOD	3.25	4.69	<LOD	125	<LOD	145	<LOQ
sheep-68	<LOD	<LOD	<LOD	<LOD	<LOD	61	<LOD	160	<LOQ
sheep-69	<LOD	<LOD	<LOD	<LOD	<LOD	<LOD	<LOD	97	<LOQ
sheep-70	<LOD	<LOD	<LOD	<LOD	<LOD	<LOD	6.1	<LOQ	<LOQ
sheep-71	1.87	5.13	<LOD	<LOD	<LOD	86.5	<LOD	221	<LOQ
sheep-72	<LOD	<LOD	<LOD	<LOD	<LOD	<LOD	<LOD	<LOQ	<LOQ
sheep-73	<LOD	<LOD	<LOD	<LOD	<LOD	<LOD	<LOD	<LOQ	<LOQ
sheep-74	<LOD	<LOD	<LOD	<LOD	<LOD	98.5	<LOD	101	<LOQ
sheep-75	<LOD	<LOD	<LOD	<LOD	<LOD	<LOD	<LOD	<LOQ	<LOQ
sheep-76	<LOD	<LOD	<LOD	2.78	<LOD	<LOD	<LOD	114	<LOQ
sheep-77	0.73	3.65	<LOD	<LOD	<LOD	445	<LOD	418	<LOQ
sheep-78	<LOD	<LOD	<LOD	<LOD	<LOD	<LOD	<LOD	<LOQ	<LOQ
sheep-79	<LOD	<LOD	<LOD	<LOD	<LOD	<LOD	<LOD	<LOQ	<LOQ
sheep-80	<LOD	<LOD	<LOD	<LOD	<LOD	147	<LOD	321	<LOQ
sheep-81	<LOD	<LOD	<LOD	<LOD	<LOD	<LOD	<LOD	<LOQ	<LOQ
sheep-82	<LOD	<LOD	2.87	5.13	<LOD	89.8	<LOD	158	<LOQ
sheep-83	4.01	<LOD	<LOD	<LOD	<LOD	<LOD	<LOD	<LOQ	<LOQ
sheep-84	<LOD	<LOD	<LOD	<LOD	<LOD	<LOD	<LOD	225	<LOQ
sheep-85	<LOD	<LOD	<LOD	<LOD	<LOD	94.3	30.8	335	<LOQ
sheep-86	<LOD	<LOD	<LOD	<LOD	<LOD	159	<LOD	<LOQ	<LOQ
sheep-87	<LOD	<LOD	0.84	<LOD	<LOD	143	<LOD	114	<LOQ

Sample	AFG2	AFG1	AFB2	AFB1	OTB	ZEA	OTA	DON	STER
sheep-88	<LOD	<LOD	<LOD	<LOD	<LOD	<LOD	<LOD	148	<LOQ
sheep-89	<LOD	<LOD	<LOD	5.64	<LOD	257	<LOD	156	<LOQ
sheep-90	0.84	<LOD	<LOD	<LOD	<LOD	<LOD	<LOD	274	2.2
sheep-91	<LOD	<LOD	<LOD	<LOD	<LOD	306	<LOD	315	<LOQ
sheep-92	<LOD	<LOD	2.96	<LOD	<LOD	<LOD	<LOD	<LOQ	<LOQ
sheep-93	<LOD	<LOD	<LOD	<LOD	<LOD	59	<LOD	425	<LOQ
sheep-94	2.1	<LOD	<LOD	<LOD	<LOD	258	<LOD	<LOQ	3.1
sheep-95	<LOD	<LOD	0.77	<LOD	<LOD	<LOD	<LOD	332	<LOQ
sheep-96	0.9	<LOD	<LOD	<LOD	<LOD	225	<LOD	214	4.1
sheep-97	<LOD	<LOD	<LOD	<LOD	<LOD	<LOD	<LOD	97	3.6
sheep-98	<LOD	<LOD	1.33	<LOD	<LOD	319	<LOD	115	<LOQ
sheep-99	<LOD	4.83	<LOD	<LOD	<LOD	<LOD	<LOD	107	<LOQ
sheep-100	<LOD	<LOD	<LOD	5.81	<LOD	101	<LOD	81	<LOQ
*LOD (ug/kg)	0.63	2.0	0.63	2.0	5.0	42.0	5.0		
*LOQ (ug/kg)	1.26	4.0	1.26	4.0	10.0	84.0	10.0	74.0	0.8

Italics: value >LOD <LOQ

Table S10. Correlation matrix (p=0.05).

	AFG2	AFG1	AFB2	AFB1	OTB	ZEA	OTA	DON	STER
**AFG2**	1								
**AFG1**	0.2528*	1							
**AFB2**	0.0205	0.0155	1						
**AFB1**	-0.0328	0.0657	0.1736*	1	-				
**OTB**	-	-	-	-	-				
**ZEA**	0.1149*	0.1594*	0.1040*	0.0509	-	1			
**OTA**	-0.0368	-0.0283	0.047	-0.0387	-	0.0686	1		
**DON**	0.0441	0.1025*	0.0358	0.0632	-	0.3129*	-0.058	1	
**STER**	-0.0336	-0.0137	0.0051	-0.0297	-	-0.0083	0.0158	-0.0002	1
